# Identification of Diverse Lipid Droplet Targeting Motifs in the PNPLA Family of Triglyceride Lipases

**DOI:** 10.1371/journal.pone.0064950

**Published:** 2013-05-31

**Authors:** Sricharan Murugesan, Elysa B. Goldberg, Eda Dou, William J. Brown

**Affiliations:** Department of Molecular Biology and Genetics, Cornell University, Ithaca, New York, United States of America; The University of Queensland, Australia

## Abstract

Members of the Patatin-like Phospholipase Domain containing Protein A (PNPLA) family play key roles in triglyceride hydrolysis, energy metabolism, and lipid droplet (LD) homoeostasis. Here we report the identification of two distinct LD targeting motifs (LTM) for PNPLA family members. Transient transfection of truncated versions of human adipose triglyceride lipase (ATGL, also known as PNPLA2), PNPLA3/adiponutrin, or PNPLA5 (GS2-like) fused to GFP revealed that the C-terminal third of these proteins contains sequences that are sufficient for targeting to LDs. Furthermore, fusing the C-termini of PNPLA3 or PNPLA5 confers LD localization to PNPLA4, which is otherwise cytoplasmic**.** Analyses of additional mutants in ATGL, PNPLA5, and Brummer Lipase, the *Drosophila* homolog of mammalian ATGL, identified two different types of LTMs. The first type, in PNPLA5 and Brummer lipase, is a set of loosely conserved basic residues, while the second type, in ATGL, is contained within a stretch of hydrophobic residues. These results show that even closely related members of the PNPLA family employ different molecular motifs to associate with LDs.

## Introduction

Lipid droplets (LDs) are evolutionarily conserved organelles that store dietary fats in the form of triacylglycerols (TAG) and sterol esters [1]–[4]. LDs consist of a neutral lipid core surrounded by a phospholipid monolayer with a wide variety of associated proteins. They are dynamic organelles existing in a continuum of growth and degradation in response to the metabolic and nutrient conditions of the cell or organism. Lipolysis, or the breakdown of stored TAGs, has been extensively studied in mammalian adipocytes due to their central role in regulating fat and energy metabolism [5]–[7]. Recent studies have shown that the Patatin-like Phospholipase Domain containing Protein A (PNPLA) family contains members that play important roles in TAG metabolism and LD homeostasis [6], [8]. The human genome encodes nine family members (PNPLA1-9), five that are closely related (PNPLA1-5), and of these, three are constitutively associated with LDs, adipose triglyceride lipase (ATGL, or PNPLA2), PNPLA3 (or adiponutrin), and PNPLA5 (or GS2-like) [9]–[11]. All family members contain a conserved N-terminal patatin domain that harbors an α/β hydrolase fold with a GXSXG-lipase motif, which is part of a Ser/Asp catalytic dyad required for enzymatic activity [5]. In contrast to their N-termini, PNPLA1-5 are variable at their C-termini but with regions of conserved residues.

ATGL, the most characterized family member, is the rate-limiting enzyme involved in TAG hydrolysis [6], [12]. It is highly expressed in adipose tissue, heart, and skeletal muscle [9], [13]. Transfection of ATGL in cells revealed colocalization with LDs and resulted in decreased TAG storage, decreased number and size of LDs, and increased liberation of free fatty acids (FFAs) [10], [13]–[15]. Reduction of protein levels using siRNA resulted in LDs with larger volumes [15]. Similarly, ATGL knockout mice had increased TAG content in multiple tissues, including adipose, heart, skeletal muscle, liver, kidneys and testis, and they exhibited cardiac dysfunction and early death [16]. Reduced release of FFAs led to defective cold adaptation and caused the mice to rely on glucose stores resulting in increased glucose tolerance and insulin sensitivity. Thus, ATGL is an important TAG lipase, playing roles in lipid catabolism of cellular fats and maintaining energy homeostasis. In the current model for stimulated lipolysis, ATGL is activated by another LD-associated protein, CGI-58 (also known as α/β hydrolase domain-containing protein 5), to hydrolyze TAG to diacylglycerol (DAG) [5], [17], [18], which is then hydrolyzed by Hormone Sensitive Lipase (HSL) to produce monoacylglycerol [19]. Conversely, G0/G1 switch gene-2 protein (G0S2) can inhibit ATGL-mediated lipolysis [20]. Further evidence for the importance of ATGL in LD degradation comes from identification of C-terminal truncation mutations in human ATGL found in patients with a form of Neutral Lipid Storage Disease with Myopathy (NLSDM) [21]–[25]. Loss of the C-terminal region due to a premature stop codon resulted in an enzymatically active protein but with low LD–associated lipase activity leading to defective TAG catabolism. As a consequence, patients with NLSDM accumulate TAGs in heart, skeletal muscle, and liver, among other tissues [6], [21], [22].

The results with NLSDM cells suggest that ATGL, and by extension PNPLA3 and PNPLA5, must physically associate with LDs to efficiently carry out their functions. Proteins use a variety of sequence motifs to bind LDs [4], [26]; however, the molecular mechanisms that target PNPLA family members to LDs are unknown. Previous studies established that ATGL is constitutively localized to LDs, unlike HSL that translocates to LDs upon signal-induced phosphorylation [15], [27]. Evidence from truncation mutations in NLSDM patients and other studies point to targeting information residing within the C-terminal region [28], [29]. Also, LD localization is not dependent on enzymatic activity because point mutations in the catalytic site serine have no effect on localization [15]. Another study has suggested that ATGL is delivered to LDs via components of the COPII and COPI membrane trafficking machinery [30]. Far less is known about the sequence or motifs within PNPLA3 and PNPLA5 that are utilized for targeting to LDs, or if these are conserved within the family. In this study, we conducted molecular dissection and mutational analyses of the LD-associated PNPLA family members to identify amino acid sequences or motifs that are responsible for their association with LDs.

Here we show that the C-terminal third of all three LD-associated family members is sufficient for LD association, and within these domains two different molecular motifs are employed. First, human PNPLA5 and Brummer Lipase, the *Drosophila* homolog of human ATGL [31], both contain a C-terminal LD targeting motif (LTM) consisting of loosely conserved basic residues, which likely form an amphipathic helix. This amphipathic helix could facilitate the association of PNPLA5 and Brummer Lipase with the negatively charged LD surface [32], thus aiding their ability to interact with substrates. Second, ATGL contains a 40 residue hydrophobic stretch in the C-terminus that is important for LD association. Thus, the PNPLA family uses (at least) two different molecular motifs for association with LDs.

## Results

### The C-terminal Third of Human PNPLA5 Contains Sequences Required for LD Localization

**Figure 1:**
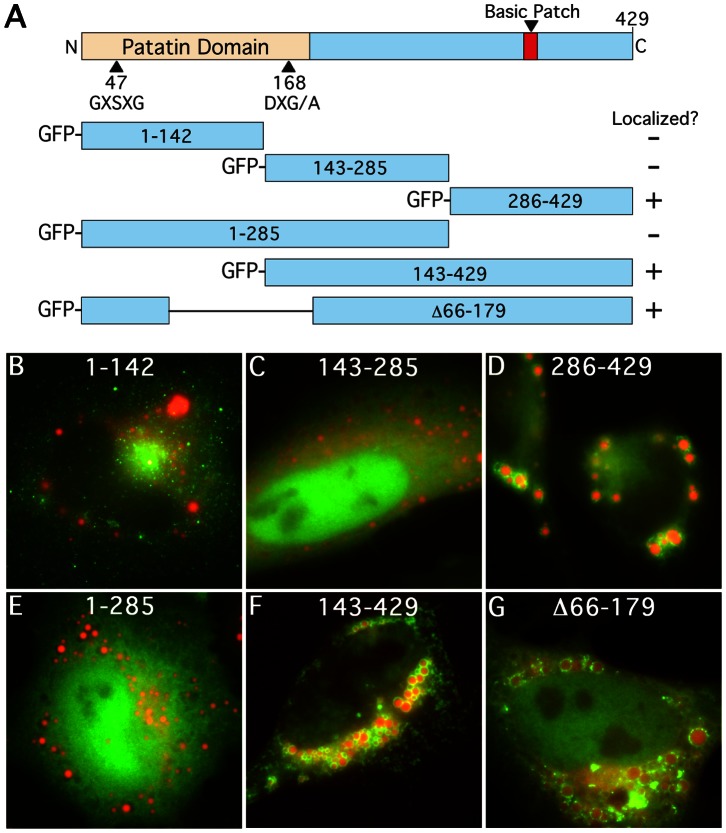
GFP-tagged proteins containing the C-terminal third of human PNPLA5 localize to LDs. (A) Schematic of the domain structure of full length PNPLA5 highlighting the N-terminal catalytic dyad within the Patatin domain, the C-terminal basic patch region, and all truncation mutants generated. HeLa cells were treated overnight with OA, transfected with the indicated constructs for 24 h, fixed and stained with LipidTox Red, and then analyzed by fluorescence microscopy. N-terminal GFP-tagged PNPLA5 constructs missing the C-terminal third of the protein were found in the cytoplasm or nucleus (B, C, and E), whereas constructs containing the C-terminal third of PNPLA5 (residues 286–429), or one lacking a portion of the patatin domain localized to LipidTox stained LDs (D, F, and G). Bars, 5 μm.

To identify sequences in PNPLA family members that are important for LD targeting, we used PNPLA5 as a model because it is highly conserved in its C-terminus between species. Human PNPLA5 is a 429 residue protein that contains an N-terminal patatin α/β hydrolase domain and an uncharacterized C-terminal domain (Fig. 1A). We have previously observed that PNPLA5 is capable of reducing stored TAGs in LDs (Murugesan et al., unpublished data). When expressed in HeLa cells, it localizes to LDs, although to a lesser extent (∼30–40% of all cells) than ATGL, PNPLA3, or its mouse homolog (∼100% of all cells) indicating the potential for diverse LD targeting mechanisms within this lipase family (Fig. S1B, D, first bar). To identify regions of PNPLA5 that are important for LD association, truncation mutants were N-terminally fused to GFP (Fig. 1A), expressed in HeLa cells fed oleic acid (OA) to induce LD formation, and cells were analyzed by fluorescence microscopy. The results showed that any construct missing the C-terminal third of the protein was found in the cytoplasm and/or nucleus (Fig. 1B, C, E), whereas any construct containing the C-terminal third localized to LDs, e.g., PNPLA5(286–429) (Fig. 1D). A shorter PNPLA5 cDNA (Δ66–179), which is missing residues 66–179 within the patatin-α/β hydrolase domain, also showed robust LD localization (Fig. 1G). As expected for a construct that cannot bind to LDs, overexpression of PNPLA5(1–286) did not reduce LD size, but instead produced a slight increase, even though the catalytic domain was intact (Fig. S1A). This apparent dominant-negative affect could be a consequence of decreased TAG hydrolysis by other LD proteins, inappropriate LD fusion (or inhibition of fragmentation), or other unknown affects. These results demonstrate that the C-terminal third of PNPLA5 (amino acids 286–429) contains residues that are required for LD association.

The reason for the cell-autonomous LD localization behavior of PNPLA5 is unclear. Another LD-associated lipase, hormone sensitive lipase (HSL), is responsible for hydrolyzing diacylglycerol to monoacylglycerol in response to hormone stimulation in adipocytes, and its association with LDs is regulated by protein kinase A (PKA)-dependent phosphorylation [33], [34]. To see if the association of PNPLA5 with LDs is similarly regulated by PKA phosphorylation, cells expressing GFP-PNPLA5 were treated with agents to stimulate (forskolin) or inhibit (H89, ErkII) this pathway. The results showed that none of these treatments affected the localization of PNPLA5 (Fig. S1B, C).

### Residues 340–364 of PNPLA5 Contain Information Required for LD Localization

**Figure 2:**
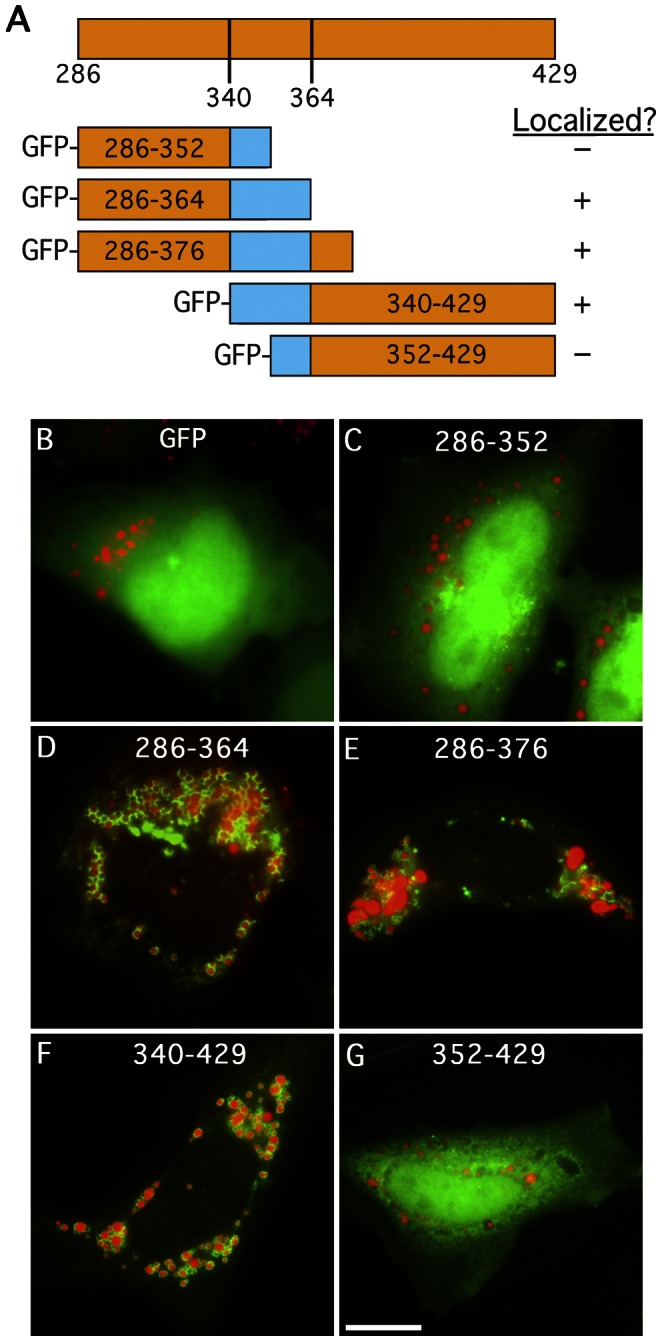
Additional truncation mutants of PNPLA5 identify a 25 amino acid domain necessary for LD localization. (A) Schematic of the C-terminal constructs fused to GFP. HeLa cells were treated overnight with OA, transfected with the indicated constructs for 24 h, fixed and stained with LipidTOX Red. Cells were then analyzed by fluorescence microscopy to observe LD localization as in Figure 1. Constructs containing residues 340–364 of PNPLA5 localized to LipidTox stained LDs (D, E, and F), whereas those missing a portion of this sequence or a control expressing GFP alone, remained in the cytoplasm (B, C, and G). Bars, 5 μm.

To more precisely define the LTM of PNPLA5, additional truncation mutants of the C-terminal fragment (residues 286–429) were generated and N-terminally fused to GFP (Fig. 2A). Starting from the C-terminus, removal of residues up to 364 had no appreciable effect on LD binding: constructs PNPLA5(286–364) and PNPLA5(286–376) (Fig. 2D, E). In contrast, removal of residues to 353, PNPLA5(286–352), prevented LD association (Fig. 2C) and resulted in cytoplasmic localization comparable to a control construct expressing GFP alone (Fig. 2B). In the opposite direction, deletion of N-terminal residues to 352, PNPLA5(352–429), also resulted in cytoplasmic localization (Fig. 2G). In contrast, a smaller N-terminal truncation, PNPLA5(340–429), was localized to LDs (Fig. 2F). These results indicate that amino acids flanking residue 352 and extending from 340–364 are important for targeting to LDs and/or for maintenance of important structural elements.

**Figure 3:**
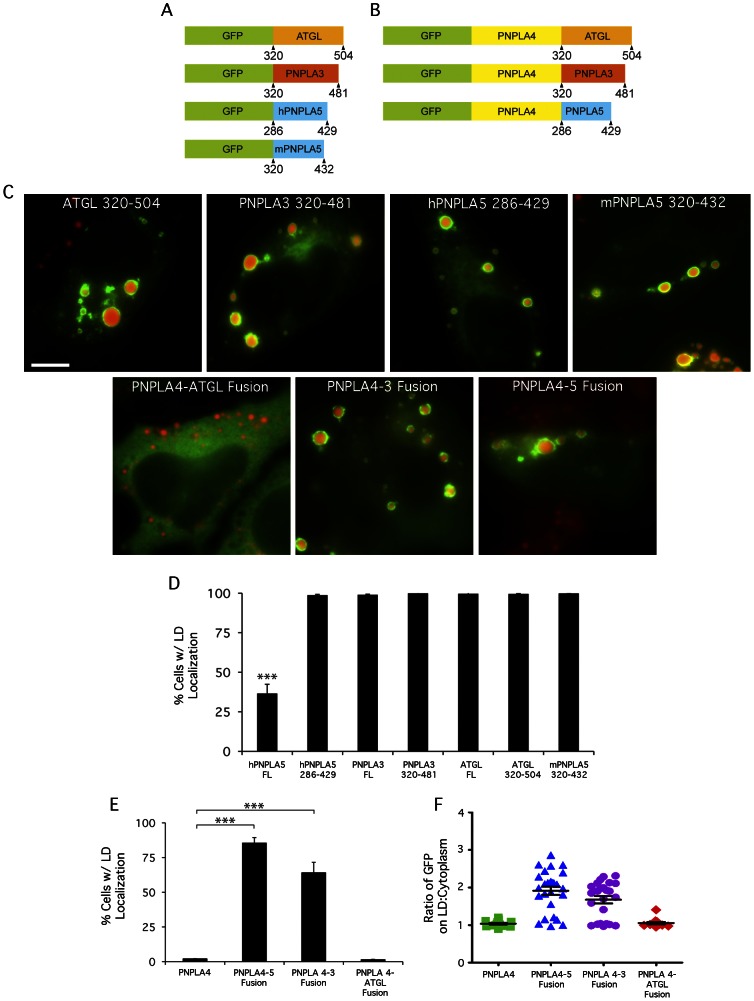
The C-terminal domains of other PNPLA family members are important for LD localization. (A, B) Schematic depicting PNPLA family C-terminal domains N-terminally fused to GFP or PNPLA4. HeLa cells were treated overnight with OA, transfected with the indicated constructs for 24 h, fixed and stained with LipidTOX Red, and analyzed by fluorescence microscopy to observe LD localization. (C, top panels) PNPLA family C-terminal domains fused to GFP localize to LDs. (C, bottom panels) Fusing C-terminal domains of PNPLA3 and PNPLA5, but not ATGL, confers LD localization to PNPLA4. The number of cells with LD surface localization in a given population was quantified as a percentage of GFP-transfected cells by scoring cells that displayed ‘rings’ of GFP signal surrounding Lipidtox stained LDs. Cells lacking LDs were not scored. To account for inherent variations in LD size/number per cell, ≥300 cells were scored per condition in three independent experiments. These results, quantified in D and E, are plotted as means ± SE (≥3 experiments/condition, ≥300 cells counted/experiment, ***, p<0.0001). (F) Quantitation of fluorescence intensity on LD surface:cytoplasm ratio from line plots (n≥15 LDs and cells/condition). FL = full length, Bar, 5 μm.

The extent of LD association of truncation mutants was determined by measuring the percentage of cells with LD-bound GFP-tagged constructs. Whereas full-length PNPLA5 was able to associate with LDs in 30–40% of cells, the C-terminal fragment, PNPLA5(286–429), localized to LDs in nearly all cells (Fig. 3A, C, D), suggesting that the N-terminus of PNPLA5 may contain residues/domains that negatively regulate its association with LDs.

### C-terminal Domains of Other PNPLA Family Members are Important for LD Targeting

We next examined if other PNPLA family members contain C-terminal LTMs (Fig. 3A). Previous studies have suggested that the C-terminal region of ATGL is important for LD targeting [23], [25], which we confirmed using a construct that lacks the entire patatin domain and contains only the C-terminal residues 320–504, ATGL(320–504) (Fig. 3C, D). Similarly, a construct containing the C-terminal third of PNPLA3, or mouse PNPLA5 tagged with GFP localized to LDs (Fig. 3C, D). To provide further evidence that the C-terminal domains are important for LD binding, we asked if LD targeting could be conferred to PNPLA4 by appending the C-terminal domains of ATGL, PNPLA3, or PNPLA5 (Fig. 3B). PNPLA4 contains a conserved N-terminal patatin domain but essentially lacks all C-terminal residues found in the other family members, and did not localize to LDs (Fig. 3E) [35]. The fusion constructs created were GFP-full length PNPLA4 with ATGL(320–504) (PNPLA4-ATGL), PNPLA3(320–481) (PNPLA4-3), and PNPLA5(286–429) (PNPLA4-5) (Fig. 3B). The results showed that PNPLA4-3 and PNPLA4-5 fusions were efficiently associated with LD (Fig. 3C, E). As expected, we observed that expressed constructs do not always associate exclusively with LDs even in cases where 100% of cells have LD localization, i.e., there are both LD and cytoplasmic pools of the enzymes. To quantify the percent of expressed protein present on LDs in comparison to the cytoplasm, we used fluorescence intensity line plots, which showed that the LD:cytoplasm ratio of the PNPLA4-3 and PNPLA4-5 fusions were significantly higher than PNPLA4 (Fig. 3F). Oddly, the fusion construct with the C-terminal domain of ATGL, PNPLA4-ATGL, did not localize to LDs suggesting that this protein may be misfolded, or the putative LTM may be masked by PNPLA4 or not presented in the appropriate structural context.

### Identification of a Basic Patch LTM in PNPLA5

**Figure 4:**
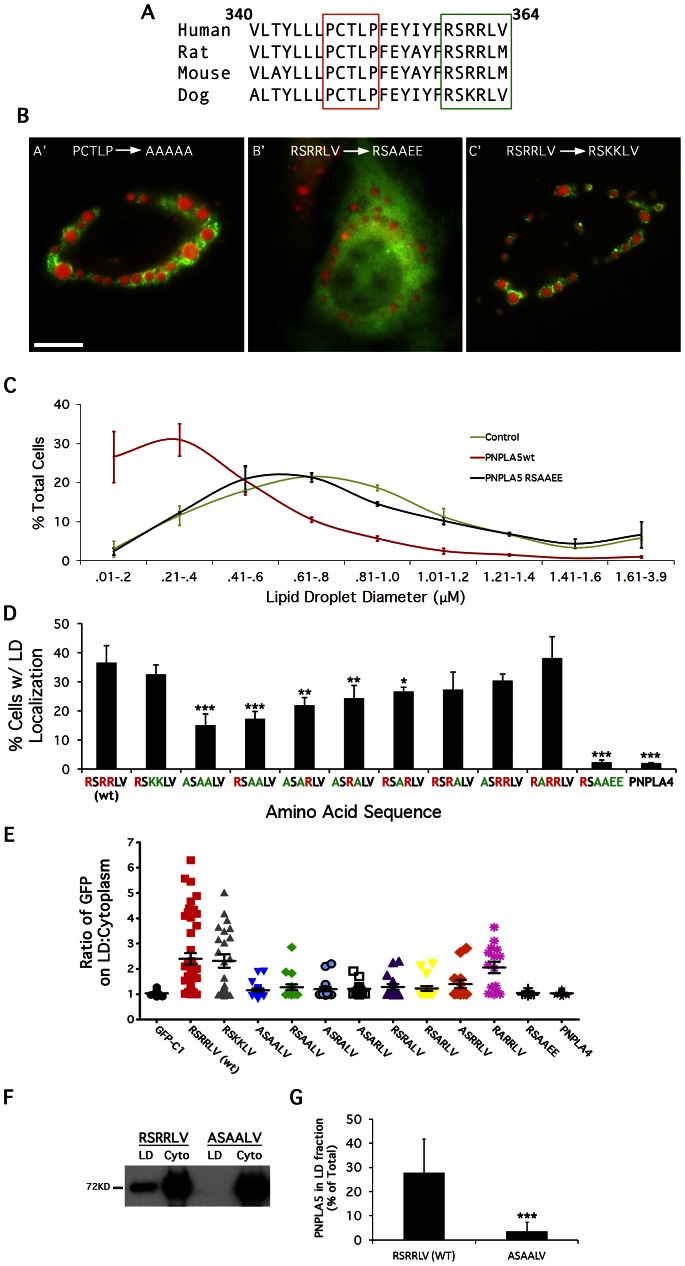
Alterations in the basic charge LTM of PNPLA5 abolish LD localization. (A) Comparison of amino acid sequences 340–364 of PNPLA5 between several species. Boxed in red are the conserved proline-rich and arginine-rich domains. (B) HeLa cells were treated overnight with OA, transfected with the indicated constructs for 24 h, fixed and stained with LipidTOX Red. Cells were then analyzed by fluorescence microscopy and scored for LD localization. GFP-PNPLA5(A_347_AAAA_352_) and GFP-PNPLA5(K_360_KLV_363_) localized to LDs, whereas GFP-PNPLA5(A_360_AEE_363_) was cytoplasmic (quantified in D). Bar, 5 μm. (C) Quantitation of LD diameters showed that overexpession of wildtype GFP-PNPLA5 reduced LD size, whereas LDs in cells over-expressing GFP-PNPLA5(A_360_AEE_363_) were similar to those in control cells. (D) Different combinations of arginines in the LTM were mutated within full length GFP-tagged PNPLA5 and LD localization was observed. No single arginine was critical for LD association but removal of each one incrementally reduced LD binding. Data are plotted as means ± SEM; ≥3 experiments/condition; ≥300 cells counted/experiment; ***indicates p<0.0001, **indicates p<0.001, *indicates p<0.05 compared to wildtype (RSRRLV). (E) Quantitation of fluorescence intensity on LD surface:cytoplasm ratio from line plots (n≥15 LDs and cells/condition). (F) HeLa cells were treated as in ‘B’ except transfections were followed by cell fractionation. Wildtype PNPLA5 (RSRRLV), but not the mutant lacking all three arginines in the basic patch (ASAALV), was enriched on LDs as shown by western blotting of isolated LD fractions in comparison to pooled cytoplasmic fractions (cyto); quantitation in (G). Data are plotted as means± SEM, n = 3.

PNPLA5 homologs in various species exhibit significant sequence conservation within amino acids 340–364 (Fig. 4A). Two sequences in particular were interesting: a proline knot-like motif that has similarity to a LD targeting domain of oleosin and the core proteins of hepatitis C virus (HCV) and GB Virus-B [36]–[39], and an arginine-containing motif that could serve as a basic patch for binding to negatively charged phospholipids on the LD surface. To determine if these are important for LD association, site-directed mutagenesis was performed on these motifs within full-length GFP-tagged PNPLA5: wild type PNPLA5(P_347_CTLP_352_) was mutated to PNPLA5(A_347_AAAA_352_) to remove the putative proline knot or to PNPLA5(A_360_AEE_363_) to completely alter the charges. Interestingly, PNPLA5(A_347_AAAA_352_) retained LD localization, whereas PNPLA5(A_360_AEE_363_) was cytoplasmic (Fig. 4B).

Since PNPLA5(A_360_AEE_363_) was unable to localize to LDs, we hypothesized that overexpressing this mutant would be ineffective at catalyzing TAG hydrolysis and reducing the size of LDs *in* *vivo*. HeLa cells were fed with OA overnight, transfected with the GFP-fusion constructs for 24 h, fixed, and diameters of LDs were analyzed by fluorescence microscopy. As expected, overexpressing wild type PNPLA5 caused a reduction in LD diameter, relative to control cells [35], but the PNPLA5(A_360_AEE_363_) construct had no such effect (Fig. 4C).

### Alterations in the Basic Patch LTM of PNPLA5 Abolish LD Targeting

The above results suggest that the conserved arginines or positively charged amino acids from residues 358–361 are important for LD association. To determine if any particular arginine (or charge) was important, we made more conservative alterations by replacing all combinations of the arginines within residues 358–361 with alanines. These changes were made in full-length GFP-tagged PNPLA5, which was expressed in HeLa cells previously fed OA to form LDs. The results showed that no particular arginine was critical but that removal of any one incrementally reduced LD association, such that alteration of all three arginines reduced LD association to background levels (Fig. 4D, E). As a more sensitive measure of LD localization, differences in subcellular localization were measured by the ratio between the fluorescence intensity at the LD surface and the cytoplasm. These fluorescence intensity line plots confirmed that only constructs with three positively charged residues had a similar LD:cytoplasm ratio distribution compared to wildtype PNPLA5 (Fig. 4E). These results were consistent regardless of the type of neutral lipid dye used, LipidTox (Fig. 4), Oil Red O (Fig. S1D), or BODIPY (data not shown). To better determine the fraction of total PNPLA5 associated with LDs, we isolated LDs from OA-fed cells using sucrose gradients and measured the distribution of PNPLA5 found in cytoplasmic vs. LD fractions by Western blot. We found that ∼27% of the total PNPLA5 was found in the LD fraction (Fig. 4F, G). Moreover, this approach verified our *in vivo* results by showing that PNPLA5 (ASAALV), the mutant lacking all three arginines, was not found in the LD fraction.

To determine if charge itself or some other feature of the RSRRLV basic patch residues is important for LD targeting, two of the arginines within this motif were changed to lysines, thus generating PNPLA5(K_360_KLV_363_). The results showed that PNPLA5(K_360_KLV_363_) localized to LDs (Fig. 4B) to the same extent as wild type PNPLA5 (Fig. 4D, E). PKA phosphorylation affects the localization of HSL and CGI58 [40], while AMP Kinase modifies the activity of ATGL [41]. However, mutating a putative AMP kinase phosphorylation site (S359A; RARRLV) had no effect on the localization of PNPLA5 (Fig. 4D). We conclude that the positively charged residues are necessary for PNPLA5 interaction with LDs.

### The Basic Patch LTM is Conserved in *Drosophila* Brummer Lipase

**Figure 5:**
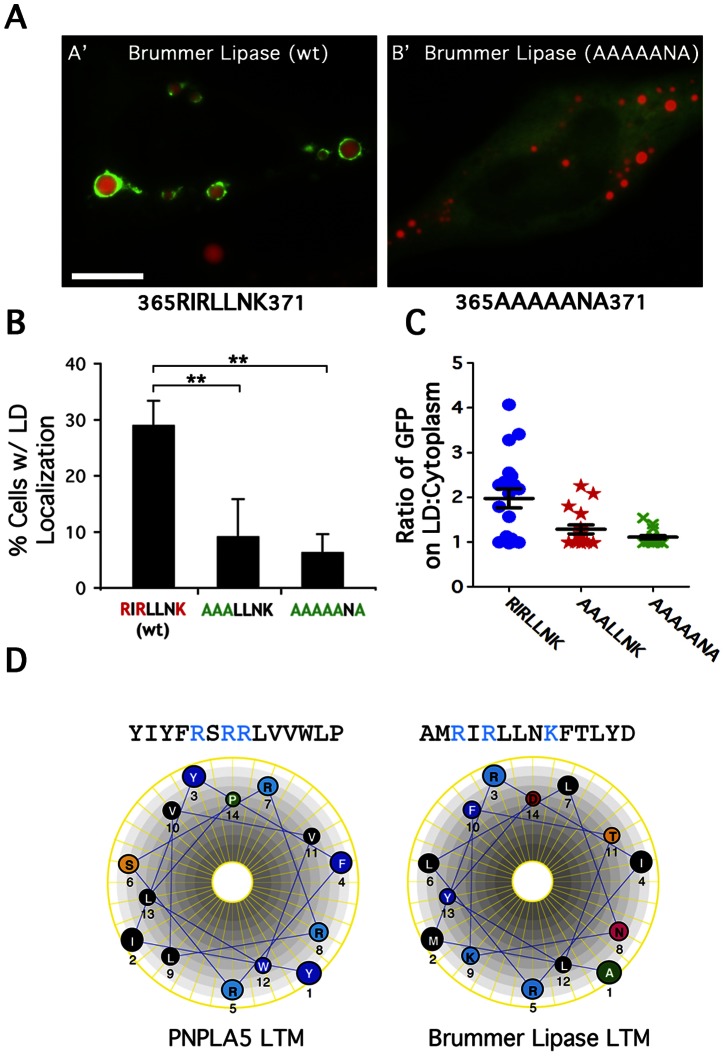
The basic patch LTM is conserved in *Drosophila* Brummer Lipase . HeLa cells were treated overnight with OA, transfected with the indicated constructs for 24 h, fixed, and stained with LipidTOX Red. Cells were then analyzed by fluorescence microscopy and scored for LD localization. (A) Wildtype *Drosophila* Brummer Lipase, the homolog of human ATGL, localized to LDs (A’) whereas mutating the three basic residues downstream of a proline knot-like motif abolished LD localization (B’). Bar, 5 μm. (B) Quantitation of LD localization. Data are plotted as means ± SEM, ≥3 experiments/condition, ≥300 cells counted/experiment; **indicates p<0.001 compared to wildtype (RIRLNK). (C) Quantitation of fluorescence intensity on LD surface:cytoplasm ratio from line plots. Wildtype Brummer lipase had a high LD:cytoplasm ratio, which was lost upon mutating the three basic residues, while mutating two residues had an intermediate effect (n≥15 LDs and cells/condition). (D) The LTM sequences of PNPLA5 (354–367) and Brummer Lipase (319–332) when projected as a helical wheel form potential amphipathic helices as determined by DNAstar.

Although the C-terminal regions of PNPLA proteins exhibit far less sequence similarity than within the N-terminal patatin domain, several members did have basic patch regions that looked similar to that of PNPLA5. In particular, we found that the *Drosophila* homolog of ATGL, Brummer Lipase, and mouse PNPLA5 also contain a basic patch downstream of a proline knot-like motif (RIRLLNK) (Fig. 5A). Previous studies have shown that targeted knock out of Brummer Lipase in flies resulted in excessive TAG storage in the fat body, and a construct missing the C-terminal half of the protein did not localize to LDs [31]. To determine if Brummer Lipase contains a basic patch LTM, we made and expressed GFP-tagged wild type and mutant versions in HeLa cells. Similar to PNPLA5, wild type Brummer lipase was targeted to LDs in ∼30% of cells; however, mutating two of the basic residues reduced targeting in half, and changing all three to alanine abolished LD targeting (Fig. 5A, B). Fluorescence intensity plots verified that the LD:cytoplasm ratios of the mutants were significantly reduced (Fig. 5C) compared to wildtype Brummer lipase. Interestingly, mutating similar arginines within residues 358–361 of mouse PNPLA5 did not reduce LD association (data not shown); however mPNPLA5 has three other basic patches that could complicate the analysis. Regardless, the basic patch LTM is conserved in at least some species.

### A Short Hydrophobic Sequence Targets ATGL to LDs

To determine if the basic patch LTMs of PNPLA5 and Brummer Lipase are conserved within the human PNPLA family, we focused our attention on the C-terminus of ATGL (Fig. S2A, S3A). Consistent with our data expressing truncated versions of ATGL (Fig. 3), other studies have shown that mutant versions of ATGL missing the C-terminal 185 amino acids, equivalent to several of the truncation mutants found in NLSDM patients, did not localize to LD droplets when expressed as YFP/GFP-tagged proteins [23], [24]. The YFP/GFP tag, use of Oil Red O as a LD stain, or overexpression could influence targeting of truncated proteins in unexpected ways. Therefore, we confirmed the importance of the C-terminal region for LD droplet association by examining the distribution of endogenous ATGL in normal and NLSDM fibroblasts by immunofluorescence. Similar to previous results using other cell types [15], in normal human skin fibroblasts, endogenous ATGL was found in a punctate distribution along the surface of LDs (Fig. S2B). In contrast, in fibroblasts from NLSDM patients, ATGL was greatly reduced on LDs and more cytoplasmic.

We hypothesized that ATGL targets LDs using C-terminal basic patch motifs, similar to those in PNPLA and Brummer Lipase. This region contains four motifs that resemble the basic patch LTMs, and three of these follow proline knot-like motifs (Fig. S2C). Therefore, we mutated several potential LTMs to determine if LD targeting was affected. We found that changing the charged residues to alanines within individual, or even all four motifs, in full length protein or a C-terminal fragment had no detectable impact on LD targeting of ATGL (Fig. S2D).

**Figure 6:**
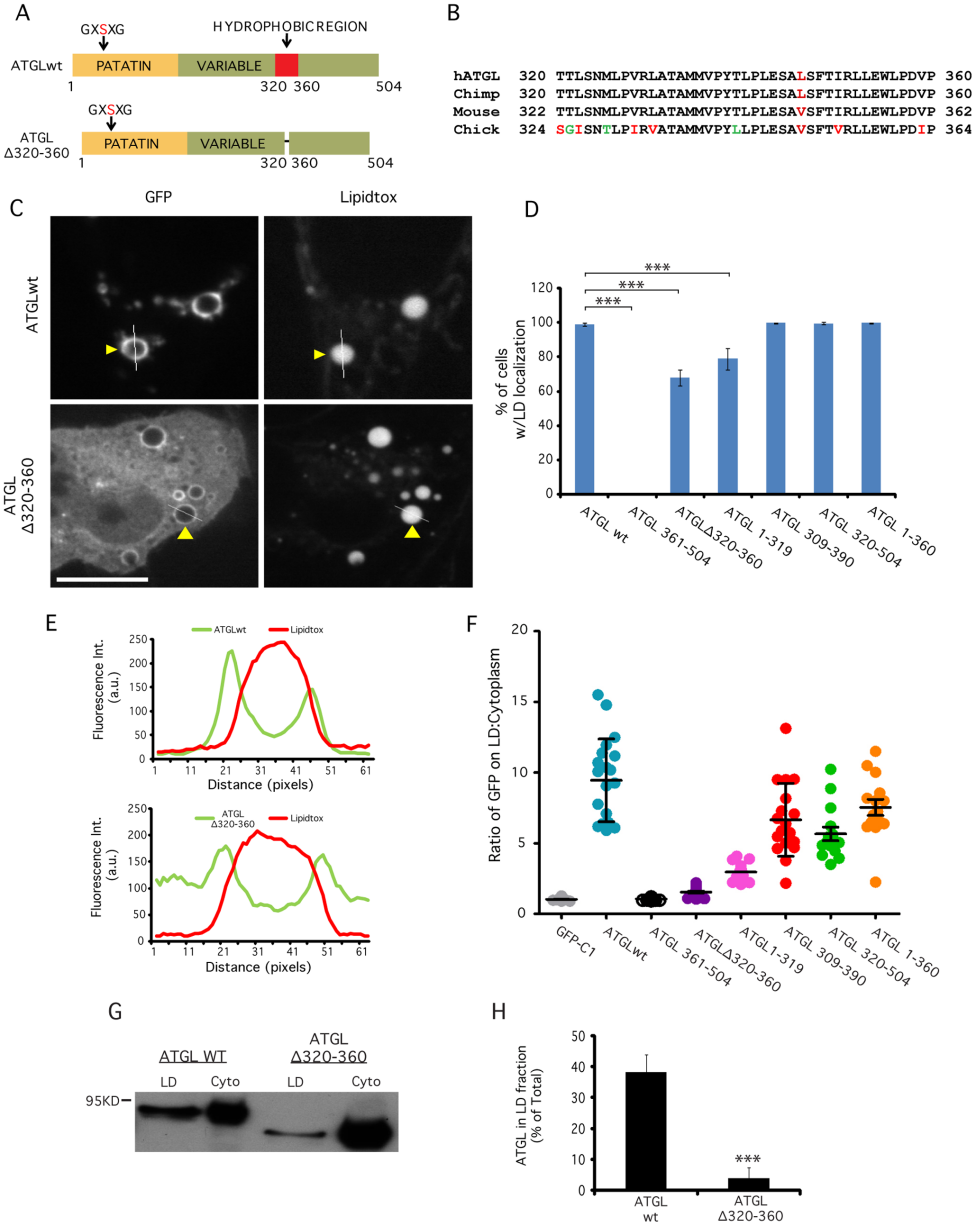
ATGL has a hydrophobic LTM. (A) A schematic diagram of ATGL depicting the putative hydrophobic LTM region within the long variable C-terminal domain. (B) Species comparison of sequences in the hydrophobic domain (residues 320–360) of ATGL orthologs. Black amino acids = identical, Red = similar, Green = unique. (C) HeLa cells were treated overnight with OA, transfected with the indicated constructs for 24 h, fixed, and stained with LipidTOX Red. Cells were then analyzed by fluorescence microscopy and scored for LD localization. Bar, 5 μm. Expression of full-length ATGL lacking the hydrophobic region (residues 320–360) in HeLa cells resulted in a decreased LD surface signal and an increased cytoplasmic signal; (D) GFP-tagged N-terminal truncations lacking residues 1–319 or a C-terminal truncation lacking residues 361–504 (but containing the hydrophobic region) were able to localize to LDs at wildtype levels; an N-terminal truncation lacking residues 1–360 (missing hydrophobic region) did not localize to LDs at all. Expressing either full-length ATGL lacking the hydrophobic residues 320–360 or a C-terminal truncation lacking residues 320–504 (found in NLSDM patients) resulted in reduced LD localization. Data are plotted as means ± SEM; ≥3 experiments/condition, ≥300 cells counted/experiment. ***indicates p<0.0001. (E) Fluorescence intensity line plot profiles from line plots in C (see arrowheads for lines). (F) Quantitation of LD surface:cytoplasm intensity ratio from line intensity plots for all ATGL constructs (plotted as mean±SEM, ≥20 LDs and cells/condition). (G) HeLa cells were treated as in ‘A’ except transfections were followed by cell fractionation. Wildtype ATGL, but not ATGL lacking the hydrophobic residues 320–360, was enriched on LDs as shown by western blotting of isolated LD fractions in comparison to pooled cytoplasmic fractions (cyto); quantitation in (H). Data are plotted as means ± SEM, n = 3.

We then switched our focus to a highly conserved hydrophobic region (residues 320–360) present in the C-terminal third of ATGL, which was previously linked to LD localization (Fig. 6A, B; Fig. S3A) [22], [25], [29], [31]. Deletion of residues 320–360, ATGL(Δ320–360), from full length ATGL resulted in an altered cellular distribution, with increased signal in the cytoplasm and a decrease on LD surfaces (Fig. 6C), including a reduced number of cells with LD localization (Fig. 6D). Fluorescence intensity plots confirmed that the LD:cytoplasmic ratio was significantly decreased in the deletion construct compared to wild type ATGL (Fig. 6E, F). Again, to determine the fraction of total ATGL found on LDs and to confirm our *in vivo* studies and line-intensity plots, we isolated LDs by sucrose density centrifugation. We found that ∼38% of total wild type ATGL, consistent with previous results [14], but only ∼4% of ATGL(Δ320–360), was found in the LD fraction (Fig. 6G, H). However, contrary to a previous report [29], deleting this hydrophobic region in ATGL only reduced the relative amount on LDs and did not abolish binding. Nevertheless, the hydrophobic region is sufficient for LD localization because an ATGL fragment containing this domain, ATGL(309–390), was able to bind LDs (Fig. 6E, Fig. S3B). In fact, any C-terminal fragment containing residues 320–360 bound to LDs, whereas those missing the hydrophobic region, ATGL(361–504), did not (Fig. 6D, F, Fig. S3B). The expression levels of GFP-tagged constructs studied above were comparable, (Fig. S4), confirming that this did not affect the differences observed in LD localization.

These results demonstrate that the hydrophobic domain is important for targeting ATGL to LDs, but also suggest that other regions may contribute as well. This suggestion is supported by experiments showing that a fragment containing the N-terminus but lacking the hydrophobic domain, ATGL(1–319), can still bind to LDs, although not as well as full-length ATGL (Fig. 6D, F). As expected, addition of the 40 hydrophobic residues, ATGL(1–360), improves overall binding to full length levels. Therefore, the N-terminal region, which also binds to the negative regulator GOS2 [20], together with the hydrophobic sequence (residues 1–360) of ATGL contribute to its targeting to LDs.

The mechanism by which these PNPLA family members are delivered to LDs is unclear. A previous study found that intracellular vesicular trafficking by COPI and COPII vesicles, which mediate Golgi-to-ER and ER-to-Golgi transport, respectively, are also involved in the delivery of ATGL to LDs [30]. For example, it was found that brefeldin A (BFA), an inhibitor of guanine nucleotide exchange factor (GEF)-activation of Arf proteins, which are required for COPI protein binding to Golgi membranes, also inhibited delivery of ATGL to LDs. In addition, recent studies found that ATGL binds to GBF1, a BFA sensitive GEF for Arf1 [28], and that knockdown of COPI components increased LD formation (or prevented their degradation) [42]. However, others found that RNAi knockdown of GBF1 had no effect on ATGL association with LDs [43], which is inconsistent with the BFA results. To explore this issue further, and to determine if other PNPLA family members might utilize the COPI-mediated delivery mechanism, cells expressing ATGL, PNPLA3, or PNPLA5 were treated with BFA before or after induction of LD formation. We confirmed that BFA treatment prevented endogenous ATGL from being recruited to LDs, but only when cells were grown in lipoprotein deficient serum (MEM+LPDS) for 72 h prior to treatment (Fig. S5A, black bars). Interestingly, BFA did not prevent overexpressed GFP-ATGL or other family members from associating with LDs (Fig. S5A, B). Moreover, BFA did not have any influence on the association of PNPLA members with LDs when cells were grown in lipoprotein containing serum (MEM+FBS) prior to treatment (Fig. S5A, B).

## Discussion

We found that the C-terminal domains of ATGL, PNPLA3, and PNPLA5 contain two different LTMs. One is a charge-dependent basic patch in human PNPLA5 and *Drosophila* Brummer Lipase. The other encompasses a hydrophobic patch in human ATGL. These results indicate that PNPLA family members interact with, or are recruited to, LDs by diverse mechanisms.

Truncation/mutational analysis of PNPLA5 and *Drosophila* Brummer Lipase identified a charge dependent LTM in the C-terminal third of the protein, where each arginine and lysine residue contributes to LD binding. When viewed as a helical wheel, these basic patches may be part of an amphipathic helix (Fig. 5D). Similar amphipathic helices are known to aid proteins in binding to negatively charged membrane surfaces [32], [44], including TIP47, which is a LD-associated perilipin family member.

Among the PNPLA family members, we observed subtle differences in the LD localization of these lipases. Wild type PNPLA5 localized to LDs in ∼35% of all cells and thus differs from the other LD localized family members since it targets LDs in a cell autonomous manner. Similarly, we found that the *Drosophila* homolog of ATGL, Brummer Lipase, also localizes to LDs in a cell autonomous fashion. One potential explanation for this phenomenon is that the localization of PNPLA5 and Brummer Lipase to LDs is regulated and dependent on the physiological state of a cell. Indeed, the localization of proteins such as HSL and CGI-58 to LDs is known to be hormonally regulated through the actions of PKA [33], [40], [45]. However, treating cells with PKA activators or inhibitors or ErkII inhibitors did not alter the localization of PNPLA5. Another possibility could involve a common group of proteins known to affect LD targeting and biology, the perilipins, whose presence on the surface of LDs is thought to prevent the access of PNPLAs to stored TAGs [7]. How these and other potential binding partners and regulatory factors control the function or (cell autonomous) localization of PNPLA5 remains uncharacterized. Other physiological states, e.g., differences in cell cycle, could be responsible for the cell autonomous localization of PNPLA5 and Brummer Lipase. Support for this conclusion is strengthened by our observation that the N-terminus of PNPLA5 may play a negative regulatory role and interfere with binding to the LD surface because the C-terminal third of PNPLA5 alone localizes to LDs more robustly than the full-length version.

The mechanism responsible for LD localization of ATGL is different from that of PNPLA5 and Brummer Lipase since it constitutively binds to LDs in all cells. Indeed, our molecular investigations of ATGL reveal that a highly conserved short hydrophobic stretch in the C-terminus of the protein is sufficient for LD localization. We should note, however, that our studies, and those cited below, have not yet demonstrated that it is the hydrophobicity of this domain, per se, that is responsible for association of ATGL with LDs. Nevertheless, our results are consistent with and extend those of Lu et al., [29] by showing that a small fragment of ATGL, extending from residues 309–390 and encompassing the hydrophobic domain of residues 320–360, is sufficient to confer LD association. Interestingly, the same region is missing in truncated forms of ATGL (Δ320–504) found in some patients with NLSDM [22]–[24]. Loss of the C-terminal region in NLSDM ATGL results in low LD–associated lipase activity leading to defective TAG catabolism [22]. Other studies expressing truncated ATGL [23], [25], show that reduced LD-associated lipase activity is partially due to the inability of ATGL (Δ320–504) to associate to LDs. Here we show that ATGL lacking residues 320–504 (equivalent to endogenous ATGL in NLSDM fibroblasts) was still able to localize to LDs, although not nearly as well as full length ATGL or C-terminal fragments containing the hydrophobic domain, confirming that ATGL’s targeting mechanism is complex and positively influenced by the N-terminus. A recent study suggests that G0S2 anchors ATGL to LDs independent of ATGL’s C-terminal lipid binding domain [46]. This observation supports our finding that ATGL (Δ320–504) is still capable of targeting LDs, presumably through G0S2, while the C-terminal hydrophobic domain might provide another mechanism of targeting, either directly or indirectly through interaction with another protein.

Regulation of LD-association and function of the PNPLA family members is complex, involves a variety of other proteins, e.g., the perilpins, and is only well understood for ATGL [47]. Perilipin1 and perilipin2 are exclusively localized to LDs while the other perilipins are present in the cytoplasm and bind to nascent LDs during rapid TAG synthesis. Interestingly, the composition of perilipins on LDs changes during adipocyte differentiation as LDs enlarge and mature. The earliest detectable LDs are coated by perilipin3 and perilipin4, but as the LDs expand, they sequentially acquire perilipin2 and eventually perilipin1 while shedding the initial LD coat [48]. Perilipins may also sequester differentially to LDs based on their neutral lipid composition [49]. Additionally, overexpression of either perilipin1 or perilipin2 increases intracellular TAG stores in LDs by reducing TAG turnover in cultured cells suggesting that they can regulate LD metabolism by shielding stored TAG from lipolytic activity [50]–[53]. In accordance with this hypothesis, ATGL fails to localize to LDs in cells lacking perilipin1. Furthermore, ATGL is recruited to LDs directly by perilipin5, which replaces perilipin1 in highly oxidative tissues such as muscle and liver [7]. Similarly, overexpression of perilipin3 protects LDs in keratinocytes and hepatocytes from degradation by retinyl esterases and lipases respectively [54], [55]. These data suggest that perilipins may serve distinct roles during various stages of LD maturation and as a result may differentially affect PNPLA localization [56]. In HeLa cells used here, perilipin2 (adipose differentiation related protein, ADRP) and perilipin3 (TIP47) localize to LDs [15], [52]. How these and other potential binding partners and regulatory factors control the function or (cell autonomous) localization of ATGL and PNPLA5 remains uncharacterized.

The association of ATGL, and probably other PNPLA family members, with LDs also involves complex interactions with other regulatory proteins, whose mechanisms are still under investigation. ATGL binds to and is activated by CGI-58 on the LD surface, but paradoxically CGI-58 is released following activation [18], [57]–[59]. Conversely, ATGL activity is negatively regulated by the G0S2 protein, which is cytoplasmic [20], [29], [60]. Several secondary structure algorithms predict that the 40 residue hydrophobic domain consists of alpha helices, which have been shown in apolipoproteins to insert into the hydrophobic environments [61], [62]. Thus, the fact that ATGL can bind to LDs in the absence of LD-associated CGI-58 or G0S2 suggests that the hydrophobic LTM, and by extension the amphiphatic LTM of PNPLA5 and Brummer Lipase, may function by directly interacting with the LD itself.

Recently, it was reported that delivery of ATGL to LDs is dependent on members of the anterograde and retrograde ER-Golgi vesicle transport machinery COPII and COPI, respectively [30]. Other studies, including mutant screens in yeast and flies, also found that COPI and trafficking proteins are involved in LD metabolism [42], [63], [64]. However, recent studies have questioned whether or not the role of COPI in LD metabolism is direct or indirect because RNAi-mediated knockdown of GBF1, the GEF for Arf1-dependent COPI vesicle formation, did not prevent ATGL from associating with LDs [43]. The reasons for the discrepancies are unclear, but our studies with BFA showed that the results depend very much on how the cells were grown prior to the experiment, i.e., we only observed BFA-inhibited endogenous ATGL recruitment when cells were grown in lipid-deficient media; also, overexpressed PNPLA family members were resistant to BFA regardless of growth conditions. These results suggest a requirement for a lipid signaling component and require further inquiry. It is clear that TAG storage in LDs increases following inhibition or loss of COPI components [30], [42], [43], but this is probably not the result of failure to deliver PNPLA family TAG lipases. Rather, inhibition of intracellular membrane trafficking may trigger a shift of fatty acids away from membrane biogenesis to storage in LDs, as seen by others [65], [66]. Thus, we suggest that COPI vesicles may not be directly involved in delivery of PNPLA family members to LDs.

Several studies, including ours, have now established that a variety of mechanisms have evolved to target proteins to LDs. For example, the perilipin family member perilipin2 (also called adipose differentiation related protein, ADRP) contains discontinuous segments with no distinguishable characteristics that target it to pre-existing LDs [67], [68]. Similar studies uncovered three hydrophobic targeting sequences in perilipin1 that confer LD association [69], [70]. Hydrophobic sequences also play important roles in LD association of caveolins, plant oleosins and Hepatitis C Virus (HCV). Furthermore, the latter two proteins also contain critical proline residues forming a “proline knot” that is thought to induce a 180° turn in the peptide backbone making it important for tertiary structure integrity [36]–[39], [44]. Additionally, the ability of perilipin3 (also called TIP47) to bind nascent LDs can be partly attributed to a C-terminal hydrophobic cleft, which when mutated causes mislocalization [71]. The heterogeneity of the known LTMs including those identified in this paper raises an important question. The LTMs thus far identified that confer LD association (hydrophobic regions, basic patches/amphipathic helices) have no obvious consensus sequences and are insufficient to explain how these proteins can discriminate amongst various membrane-bound organelles. One property that may contribute to specificity is the unique structure of a LD, which is a phospholipid monolayer surrounding a neutral lipid core with biophysical properties distinct from membrane bilayers [72]. This unique surface may allow proteins to distinguish the LD from other membranous organelles within the cell.

## Materials and Methods

### Cell Culture, Transfection, and Direct Fluorescence Microscopy

HeLa cells were maintained in minimal essential medium (MEM) with 10% Nu-Serum or Fetal Bovine Serum (FBS) and 1% penicillin/streptomycin supplements in a 37°C environment of 95% air and 5% CO_2_. Transfection of HeLa cells was performed using Lipofectamine 2000 (Invitrogen, Carlsbad, CA) as described by the manufacturer. Before experimentation, HeLa cells were grown on glass coverslips overnight.

HeLa cells were fed 60 μM oleic acid (OA) conjugated to fatty acid free bovine serum albumin (BSA) overnight to induce LD formation [73]. For fluorescence microscopy, cells were fixed with 3.7% formaldehyde in phosphate buffered saline, pH 7.4 (PBS), stained with neutral lipid dyes Oil Red O, LipidTOX, and BODIPY to stain lipid droplets [74] and mounted on slides with Vectashield (Vector Laboratories, Inc. Burlingame, CA). Cells were imaged by wide-field epifluorescence (Zeiss Axioskop 2) and Perkin-Elmer Ultraview spinning disk confocal microscope and the diameters of the lipid droplets were measured using Openlab (Improvision, Lexington, MA) software for quantitation.

### LD Isolation

Two 100 mm plates of HeLa cells (80% confluency) were fed overnight with Oleic Acid (see above), transfected with plasmids expressing either GFP-ATGL or GFP-ATGLΔ320–360, harvested by scraping in PBS and centrifuged at 2000×g for 5 min at 4°C. The following procedure was performed as close to 4°C as possible. Pellets were resuspended in homogenization buffer (0.25 M sucrose, 10 mM Tris, 1 mM EDTA, and Complete Mini Protease Inhibitors Mixture (Roche Diagnostics, Manheim, Germany)) for 10 min and lysed by needle homogenization 40 times through a 26-gauge syringe. The resulting homogenate was centrifuged for 10 min at 1000×g to remove nuclei. 400 μl of the post-nuclear supernatant (PNS) was mixed with 1 ml 2 M sucrose and pipetted into a 2 ml ultracentrifuge tube (Beckman Instruments Inc, Palo Alto, CA). Then, 300 μl amounts of 0.6 M and 0.25 M sucrose in homogenization buffer were layered on top. These gradients were centrifuged at 165,000×g for 1 h in a TLS-55 rotor. The floating LD fraction was visualized as a hazy white layer at the top of the gradient. 200 μl fractions were collected, prepared for separation by 10% SDS-PAGE, and then western blotted with rabbit anti-GFP (A. Bretscher, Cornell Univ.).

### Quantitation of LD Localization

Fixed and Lipidtox stained HeLa cells (OA treated prior to transfection with the indicated GFP-tagged constructs) were observed by fluorescence microscopy. For each experiment, images were captured under the same conditions. LD localization was measured in two ways. First, the number of cells with LD surface localization in a given population was quantified as a percentage of GFP-transfected cells by scoring cells that displayed ‘rings’ of GFP signal surrounding Lipidtox stained LDs. Cells lacking LDs were not scored. To account for inherent variations in LD size/number per cell, ≥300 cells were scored per condition in three independent experiments. Second, fluorescence intensity plot profiles were created using NIH ImageJ, as a more sensitive measure of LD localization. Differences in subcellular localization are measured by the ratio between the fluorescence intensity at the LD surface and the intensity in the cytoplasm. These ratios were measured for ∼15–40 LDs/cells per condition across three independent experiments.

### Antibodies and Generation of Constructs

Rabbit antiserum to human full length ATGL was used for immunofluorescence as described [15]. Human PNPLA5 (Accession number BC031820) and human PNPLA4/GS2 (Accession number BC020746) cDNAs were obtained from the ATCC (Manassas, VA, USA); ATGL cDNA was supplied by Catherine Jackson (Université Paris Diderot-Paris 7,Paris, France) [15]. Drosophila Brummer Lipase cDNA was obtained from the GOLD collection [75]. The BglII and KpnII sites were used to clone the full-length or fragments of human PNPLA5 into pEGFP-C1 (Clonetech, Mountain View, CA) at the 5′ and 3′ ends, respectively. The EcoRI and SalI sites were used to clone full-length or fragments of ATGL, PNPLA3, and PNPLA4 into pEGFP-C2 vector (Clonetech). Mutagenesis reactions were performed using the Quikchange II site-directed mutagenesis kit from Stratagene (La Jolla, CA). All constructs were confirmed by sequencing.

All primers and constructs are shown in Table S1 Table S2, Table S3.

## Supporting Information

Figure S1 **(A) Overexpressing GFP-PNPLA5(1–286), a construct that cannot bind to LDs, did not affect LD size when compared to untransfected HeLa cells.** (B and C) LD association of PNPLA5 and ATGL was not affected by PKA phosphorylation. HeLa cells expressing (B) GFP-PNPLA5 or (C) ATGL were treated with agents to stimulate (forskolin) or inhibit (H89, ErkII) this pathway; none of these treatments affected their localization as observed by fluorescence microscopy. (D) Overexpressing combinatorial arginine mutants of PNPLA5 while using Oil Red O instead of LipidTox to stain LDs demonstrated that alterations in the basic charge LTM of PNPLA5 reduced LD localization regardless of lipid dye used. Data are plotted as means ± SE; ≥3 experiments/condition, ≥300 cells counted/experiment. ***indicates p<0.0001.
(PDF)

Figure S2 **Examination of putative LTMs in human ATGL.** (A) Schematic of truncated form of ATGL (missing the last 185 C-terminal residues) found in NLSDM patients. (B) By immunofluorescence, endogenous ATGL in normal human skin fibroblasts was found in a punctate distribution along the surface of LDs (stained with LipidTOX Red), whereas ATGL was greatly reduced on LDs and more cytoplasmic in fibroblasts from NLSDM patients (missing last 185 residues). Bar, 5 μm. (C) Amino acid sequence of C-terminal domain in ATGL depicting four potential LTM sequences, three of which follow proline knot-like motifs (highlighted), that resemble the basic patch LTMs of PNPLA5 and Brummer Lipase. (D) HeLa cells were treated overnight with OA, transfected with the indicated GFP-tagged ATGL constructs for 24 h, fixed and stained with LipidTOX Red. Cells were then analyzed by fluorescence microscopy and scored for LD localization. Mutating the charged residues to alanine within individual, or even all four motifs, in full length (shown here) or a C-terminal fragment (data not shown), had no impact on LD localization of ATGL.
(PDF)

Figure S3 **Molecular dissection analysis of ATGL.** Cells were treated with OA overnight, transfected with GFP-tagged ATGL truncation constructs, and stained with LipidTox. (A) Domain maps of truncation constructs used to examine the role of ATGL’s hydrophobic domain on LD localization. (B) GFP-tagged C-terminal truncations of ATGL lacking residues 320–504 (found in NLSDM patients) or residues 361–504 (contains hydrophobic region) were able to localize to LDs (1^st^ and 3^rd^ rows). A GFP-tagged N-terminal truncation lacking residues 1–360 did not localize to LDs (2^nd^ row) while another that lacks 1–319 (but contains hydrophobic region) did (4^th^ row). A short GFP-tagged fragment (309–390) containing the hydrophobic domain was able to localize to LDs (5^th^ row). Bars, 5 μm.
(PDF)

Figure S4 **Expression levels of PNPLA family LTM constructs.** Lysates from cells expressing the indicated GFP-tagged constructs were subjected to western blotting with anti-GFP antibodies. (A) Western blot of GFP-tagged PNPLA5 constructs. (B) Western blot of GFP-tagged ATGL constructs. (C and D) Quantitation of results from A and B, respectively (n = 3) normalized to wildtype expression levels.
(PDF)

Figure S5 **Effect of BFA on delivery of PNPLA proteins to LDs.** Cells were grown in either 10% FBS or 10% LPDS (lipoprotein deficient serum) for 72 h and then treated with BFA either before or after incubation with OA. To examine delivery to nascent LDs, cells were incubated with 5 μM BFA for 10 min and then fed OA:BSA for 3 h in the continuous presence of BFA. To examine delivery to pre-existing LDs, cells were fed OA:BSA for 3 h to induce LD formation and then incubated with 5 μM BFA for 3 h. Cells were fixed, stained with LipidTox, and examined by fluorescence microscopy. (A) HeLa cells were either transfected to express GFP-ATGL, or stained by immunofluorescence to detect endogenous ATGL. BFA treatment reduced the association of endogenous but not expressed ATGL when cells were grown in LPDS, but it had no effect on expressed or endogenous ATGL when cells were grown in FBS. (B) GFP-tagged PNPLA3 and mPNPLA5 expressed prior to OA incubations were not affected by BFA treatment regardless of serum used. Data are plotted as means ± SE; ≥3 experiments/condition, ≥300 cells counted/experiment. ***indicates p<0.0001
(PDF)

Table SI **Primers used for generating PNPLA truncation constructs.** ATGL ATGL 320–504 EcoR1 960 (f): 5′- CCG GAA TTC ATG ACC ACC CTC TCC AAC ATG C-3′ ATGL 320-504 Sal1 1512 (r): 5′- ACG CGT CGA CTC ACA GCC CCA GGG CCC C-3′ ATGL 1-319 EcoR1 1 (f): 5′-CCG GAATTC ATG TAC GAC GCA GAG CG-3′ ATGL 1-319 Sal1 957 (r): 5′-ACGC GTCGAC GGT GTC CAG GAT GCT CTC-3′ ATGL 361-504 EcoR1 1083 (f): 5'-CCG GAA TTC GAG GAC ATC CG-3' ATGL 309-390: ACGC GTCGAC GGG CAG GTG CCT ATGL 1-360 EcoR1 1080 (r): 5'-ACG CGT CGA CGG GAA CGT CGG-3' ATGL Δ320-360 Sal1 957 (r): 5′-CG GAT GTC CTC CAG CAG GTC CGT GGG CTC-3′ ATGL Δ320-360 EcoR1 1080 (f): 5′-CCC ACG GAC CTG CTG GAG GAC ATC CG-3′ PNPLA3 PNPLA3 320-481 EcoR1 960 (f): 5′- CCG GAA TTC CCC AGG CTC GCT ACA GCA CTG -3′ PNPLA3 320-481 Sal1 1443 (r): 5′- TCA CAG ACT CTT CTC TAG TGA AAA ACT GGG -3′ PNPLA5 PNPLA5 1-142 BglII (f): 5′-TAG AGA TCT ATG GGC TTC TTA GAG GAG G-3′ PNPLA5 1-142 KpnI 428 (r): 5′-CAT GGT ACC TTA CTG GAT GAG CTC ATC GCA GGT-3′ PNPLA5 143-285 BglII 429 (f): 5′-TAC AGA TCT GCC TTG GTC TGC ACC TTA TAC-3′ PNPLA5 143-285 KpnI 855 (r): 5′-CAT GGT ACC TTA GCG TTG CTC ACA GGC AGC-3′ PNPLA5 286-429 BglII 858 (f): 5′-TAC AGA TCT TGG AAG GGG GGC CTG TCT C-3″ PNPLA5 286-429 KpnI (r): 5′-CAT GGT ACC TCA GGC CTG GTG GGT GGG-3′ PNPLA5 340-429 BglII 1020 (f): 5′-TAC AGA TCT GTG CTG ACG TAC CTG CTG C-3′ PNPLA5 352-429 BglII 1056 (f): 5′-TAC AGA TCT GAG TAC ATC TAC TTC CGC AGC-3′ PNPLA5 286-352 Kpn1 1056 (r): 5′-CAT GGT ACC TTA GAA GGG CAG TGT GCA GGG-3′ PNPLA5 286-364 Kpn1 1092 (r): 5′-CAT GGT ACC TTA CAC CAC CAA CCT TCT GCT-3′ PNPLA5 286-376 Kpn1 1128 (r): 5′-CAT GGT ACC TTA CAT CCA CCA CAA GTC CGC-3′ mPNPLA5 mPNPLA5 320-432 EcoR1 960 (f): Forward 5′-CCGGAATTCCAGAAGACTGGCCCA-3′ mPNPLA5 320-432 Sal1 1296 (r): 5′-ACGCGTCGACAGTCAGGTACGGT-3′
(DOCX)

Table S2 **Primers used for generating site-directed mutation of putative LTM.** ATGL ATGL AALL (f): 5′-GCT CTG TCC TTC ACC GCC GCC TTG CTG GAG TGG CTG-3′ ATGL AALL (r): 5′-CGG GCA GCC ACA CCA CCA ACG CTG CGC TGC GGA AG-3′ ATGL AAVQ (f): 5′-CAG GTG GAG CTG GCC GCC GTC CAG TCG CTG CC-3′ ATGL AAVQ (r): 5′-GGC AGC GAC TGG ACG GCG GCC AGC TCC ACC TG-3′ ATGL RAAAA (f): 5′-CCT GGT GAT GCG CGC CGC CGC CGC CCT GGG CAGGCA CCT GCC C -3′ ATGL RAAAA (r): 5′-GGG CAG GTG CCT GCC CAG GGC GGC GGC GGC GCG CAT CAC CAG G –3′ ATGL AAAAA (f): 5′-CAG TAC CTG GTG ATG GCC GCC GCC GCC -3′ ATGL AAAAA (r): 5′-GGC GGC GGC GGC CAT CAC CAG GTA CTG-3′ ATGL IAWMA (f): 5′-CCG AGG ACA TCG CT GGA TGG CGG AGC AGA CGG GC-3′ ATGL IAWMA (r): 5′-GCC CGT CTG CTC CGC CAT CCA CGC GAT GTC CTC GG-3′ ATGL AAVQA (f): 5′-GCC GCG TCC AGG CGC TGC CGT CCG T-3′ ATGL AAVQA (r): 5′-ACG GAC GGC AGC ACC TGG ACG CGG C-3′ PNPLA5 AAEE (f): 5′-C TTC CGC AGC GCA GCG GAG GAG GTG TGG CTG CCC GAT GTG CCG-3′ PNPLA5 AAEE (r): 5′-CGG CAC ATC GGG CAG CCA CAC CTC CTC CGG TGC GCT GCG GAA G-3′ PNPLA5 AASAA (f): 5′-CTG ACG TAC CTG CTG CTA GCC GCC TCA GCG GCC TTC GAG TAC ATC-3′ PNPLA5 AASAA (r): 5′-GAT GTA CTC GAA GGC GGC TGA GGC GGC TAG CAG CAG GTA CGT CAG-3′ PNPLA3 PNPLA3 QAAA (f): 5′- CTG CCA TTG CGA TTG TCC AGG CAG CGG CGA CAT GGC TTC CAG -3′ PNPLA3 QAAA (r): 5′-CTG GAA GCC ATG TCG CCG CTG CCT GGA CAA TCG CAA TGG CAG -3′ PNPLA5 PNPLA5 AAEE (f): 5′-C TTC CGC AGC GCA GCG GAG GAG GTG TGG CTG CCC GAT GTG CCG-3′ PNPLA5 AAEE (r): 5′-CGG CAC ATC GGG CAG CCA CAC CTC CTC CGG TGC GCT GCG GAA G-3′ PNPLA5 AASAA (f): 5′-CTG ACG TAC CTG CTG CTA GCC GCC TCA GCG GCC TTC GAG TAC ATC-3′ PNPLA5 AASAA (r): 5′-GAT GTA CTC GAA GGC GGC TGA GGC GGC TAG CAG CAG GTA CGT CAG-3′ PNPLA5 AAAAA (f): 5′-CTG ACG TAC CTG CTG CTA CGG CGG CGA GCG-3′ PNPLA5 AAAAA (r): 5′-GAT GTA CTC GAA GGC CGC TGC GGC GGC TAG-3′ PNPLA5 RSKKLV (f): 5′-CTT CCG CAG CAA AAA GTT GGT GGT GTG GCT GCC-3′ PNPLA5 RSKKLV (r): 5′-GGC AGC CAC ACC ACC AAC TTT TTG CTG CGG AAG-3′ PNPLA5 RSAALV (f): 5′-CTT CCG CAG CGC AGC GTT GGT GGT GTG GCT GCC CG-3′ PNPLA5 RSAALV (r): 5′-CGG GCA GCC ACA CCA CCA ACG CTG CGC TGC GGA AG-3′ PNPLA5 RSARLV (f): 5′-CTT CCG CAG CGC AAG GTT GGT GGT GTG GC-3′ PNPLA5 RSARLV (r): 5′-GCC ACA CCA CCA ACC TTG CGC TGC GGA AG-3′ PNPLA5 RSRALV (f): 5′-CTT CCG CAG CAG AGC GTT GGT GGT GTG GC-3′ PNPLA5 RSRALV (r): 5′-GCC ACA CCA CCA ACG CTC TGC TGC GGA AG-3′ PNPLA5 RARRLV (f): 5′-CAT CTA CTT CCG CGC CAG AAG GTT GGT GGT G-3′ PNPLA5 RARRLV (r): 5′-CAC CAC CAA CCT TCT GGC GCG GAA GTC GAT G-3′ PNPLA5 ASARLV (f): 5′-CAT CTA CTT CGC CAG CGC AAG GTT GGT GGT GTG G-3′ PNPLA5 ASARLV (r): 5′-CCA CAC CAC CAA CCT TGC GCT GGC GAA GTA GAT G-3′ PNPLA5 ASRALV (f): 5′- CAT CTA CTT CGC CAG CAG AGC GTT GGT GGT GTG G-3′ PNPLA5 ASRALV (r): 5′-CCA CAC CAC CAA CGC TCT GCT GGC GAA GTA GAT G-3′ PNPLA5 ASRRLV (f): 5′-GAG TAC ATC TAC TTC GCC AGC AGA AGG T-3′ PNPLA5 ASRRLV (r): 3′-ACC TTC TGC TGG CGA AGT AGA TGT ACT C-3′ PNPLA5 ASAALV (f): 5′-GTA CAT CTA CTT CGC CAG CGC AGC GTT GGT GG-3′ PNPLA5 ASAALV (r): 5′-CCA CCA ACG CTG CGC TGG CGA AGT AGA TGT AC-3′ Brummer Lipase BL AAAAA (f): 5′-CCA CGC CAT GGC GGC AGC AGC GGC-3′ BL AAAAA (r): 5′-GGC GCT GCT GCC GCC ATG GCG TGG-3′ BL AAAAANA (f): 5′-GGC AGC AGC GGC AAA TGC ATT CAC GCT CTA TGA C-3′ BL AAAAANA (r): 5′-GTC ATA GAG CGT GAA TGC ATT TGC CGC TGC TGC C-3'
(DOCX)

Table S3 **Primers used to generate PNPLA4 fusion constructs.** PNPLA4-cterm ATGL (f): 5′-ATG AAG CAC ATC AAC CTA TCA TTT GCA GCG-3′ (r): 5′-TCC CGG CAG CTG CGA GTA ATG TTC AAA CCA ATT TTC TTT AAG-3′ (f): 5′-CTT AAA GAA AAT TGG TTT GAA GAT TAC TCG CAG CTG CCG GGA-3′ (r): 5′-TCA CAG CCC CAG GGC CCC GAT-3′ EcoR1 (f): 5′-CCG GAA TTC ATG AAG CAC ATC AAC CTA TCAT-3′ Sal1 (r): 5′- ACG CGT CGA CTC ACA GCC CCA GGG CCC C-3′ PNPLA4-cterm PNPLA3 (f): 5′-ATG AAG CAC ATC AAC CTA TCA TTT GCA GCG-3′ (r): 5′-CTT AAA GAA AAT TGG TTT GAA CCC AGG CTC GCT ACA GCA CTG-3′ (f): 5′-CTT AAA GAA AAT TGG TTT GAA CCC AGG CTC GCT ACA GCA CTG-3′ (r): 5′-TCA CAG ACT CTT CTC TAG TGA AAA ACT GGG-3′ EcoR1 (f): 5′-CCG GAA TTC ATG AAG CAC ATC AAC CTA TCAT-3′ Sal1 (r): 5′-ACG CGT CGA CTC ACA GAC TCT TCT CTA GTG A-3′ PNPLA4-cterm PNPLA5 (f): 5′-ATG AAG CAC ATC AAC CTA TCA TTT GCA GCG-3′ (r): 5′-GTT GAG AGA CAG GCC CCC CTT TTC AAA CCA ATT TTC TTT AAG-3′ (f): 5′-CTT AAA GAA AAT TGG TTT GAA AAG GGG GGC CTG TCT CTC AAC-3′ (r): 5′-TCA GGC CTG GTG GGT GGG CCC-3′ EcoR1 (f): 5′-CCG GAA TTC ATG AAG CAC ATC AAC CTA TCAT-3′ Sal1 (r): 5′-ACG CGT CGA CTC AGG CCT GGT-3′
(DOCX)
