# Supplementary figures and images for: Identification of Diverse Lipid Droplet Targeting Motifs in the PNPLA Family of Triglyceride Lipases
Source: PLoS One. 2013 May 31;8(5):e64950. doi: 10.1371/journal.pone.0064950 (PMC3669214; doi:10.1371/journal.pone.0064950)

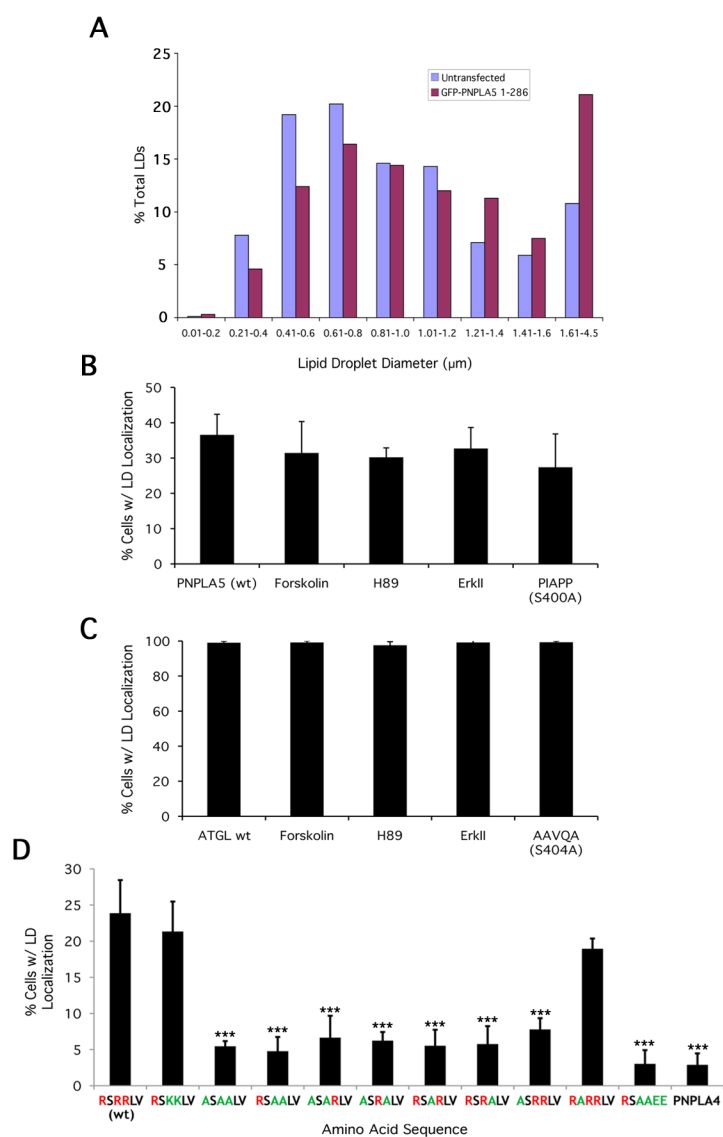

Murugesan et al.  
Supplemental Figure S1

Supplement: Figure S1 — (A) Overexpressing GFP-PNPLA5(1–286), a construct that cannot bind to LDs, did not affect LD size when compared to untransfected HeLa cells. (B and C) LD association of PNPLA5 and ATGL was not affected by PKA phosphorylation. HeLa cells expressing (B) GFP-PNPLA5 or (C) ATGL were treated with agents to stimulate (forskolin) or inhibit (H89, ErkII) this pathway; none of these treatments affected their localization as observed by fluorescence microscopy. (D) Overexpressing combinatorial arginine mutants of PNPLA5 while using Oil Red O instead of LipidTox to stain LDs demonstrated that alterations in the basic charge LTM of PNPLA5 reduced LD localization regardless of lipid dye used. Data are plotted as means ± SE; ≥3 experiments/condition, ≥300 cells counted/experiment. ***indicates p<0.0001. (PDF) [file pone.0064950.s001.pdf]

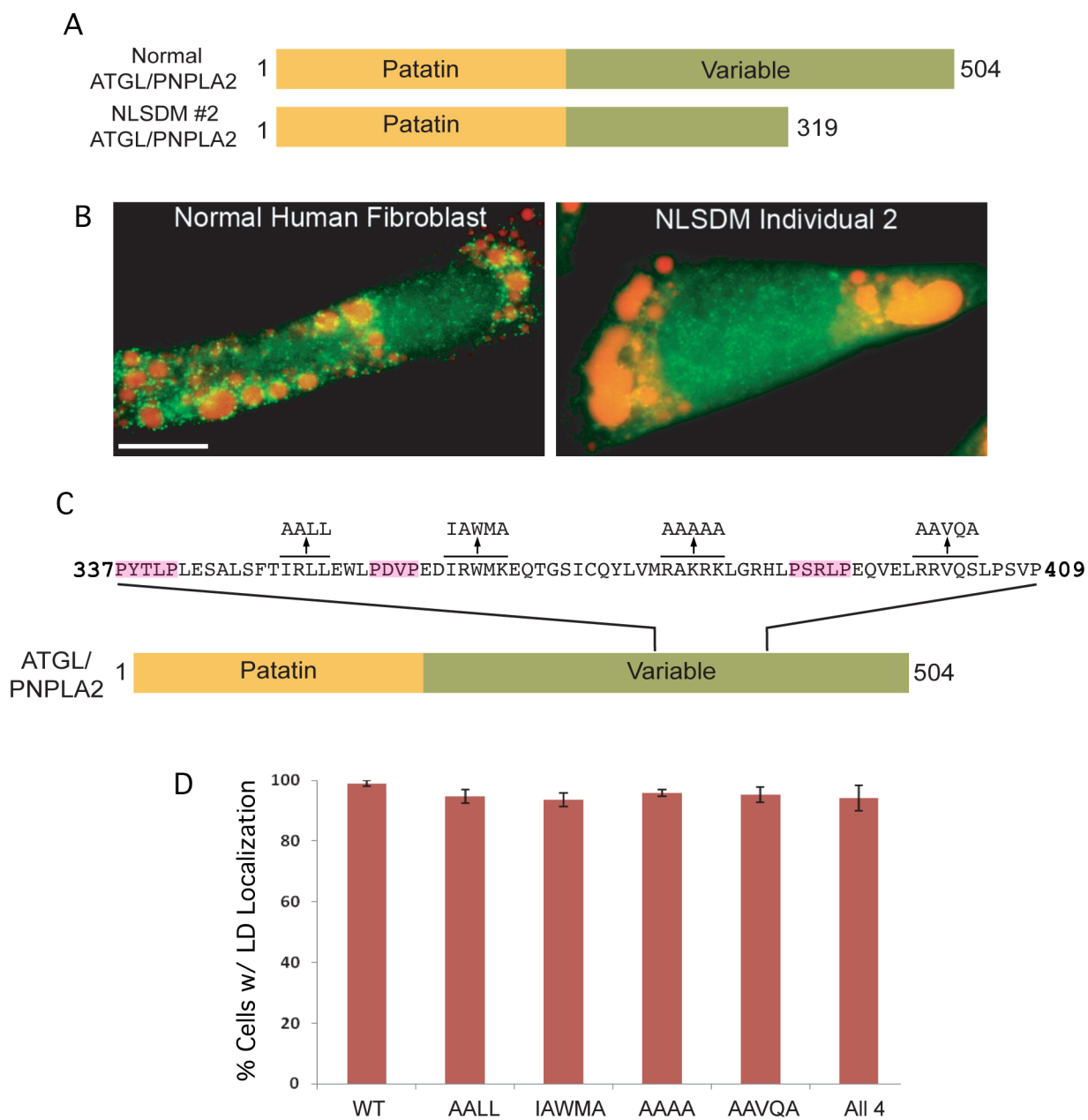

Murugesan et al.  
Supplemental Figure S2

Supplement: Figure S2 — Examination of putative LTMs in human ATGL. (A) Schematic of truncated form of ATGL (missing the last 185 C-terminal residues) found in NLSDM patients. (B) By immunofluorescence, endogenous ATGL in normal human skin fibroblasts was found in a punctate distribution along the surface of LDs (stained with LipidTOX Red), whereas ATGL was greatly reduced on LDs and more cytoplasmic in fibroblasts from NLSDM patients (missing last 185 residues). Bar, 5 μm. (C) Amino acid sequence of C-terminal domain in ATGL depicting four potential LTM sequences, three of which follow proline knot-like motifs (highlighted), that resemble the basic patch LTMs of PNPLA5 and Brummer Lipase. (D) HeLa cells were treated overnight with OA, transfected with the indicated GFP-tagged ATGL constructs for 24 h, fixed and stained with LipidTOX Red. Cells were then analyzed by fluorescence microscopy and scored for LD localization. Mutating the charged residues to alanine within individual, or even all four motifs, in full length (shown here) or a C-terminal fragment (data not shown), had no impact on LD localization of ATGL. (PDF) [file pone.0064950.s002.pdf]

A

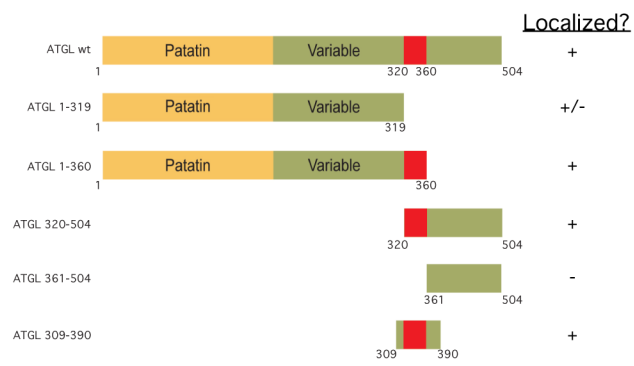

B

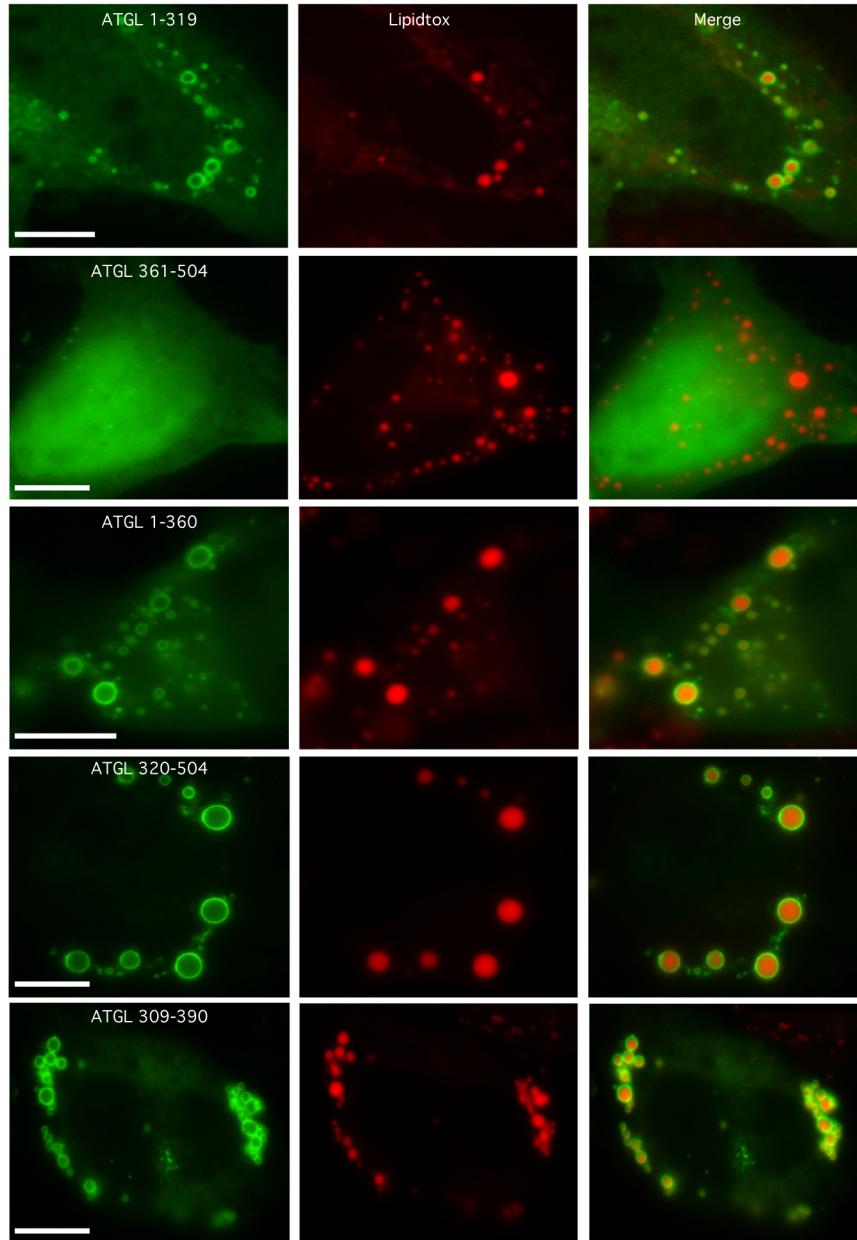

Supplement: Figure S3 — Molecular dissection analysis of ATGL. Cells were treated with OA overnight, transfected with GFP-tagged ATGL truncation constructs, and stained with LipidTox. (A) Domain maps of truncation constructs used to examine the role of ATGL’s hydrophobic domain on LD localization. (B) GFP-tagged C-terminal truncations of ATGL lacking residues 320–504 (found in NLSDM patients) or residues 361–504 (contains hydrophobic region) were able to localize to LDs (1st and 3rd rows). A GFP-tagged N-terminal truncation lacking residues 1–360 did not localize to LDs (2nd row) while another that lacks 1–319 (but contains hydrophobic region) did (4th row). A short GFP-tagged fragment (309–390) containing the hydrophobic domain was able to localize to LDs (5th row). Bars, 5 μm. (PDF) [file pone.0064950.s003.pdf]

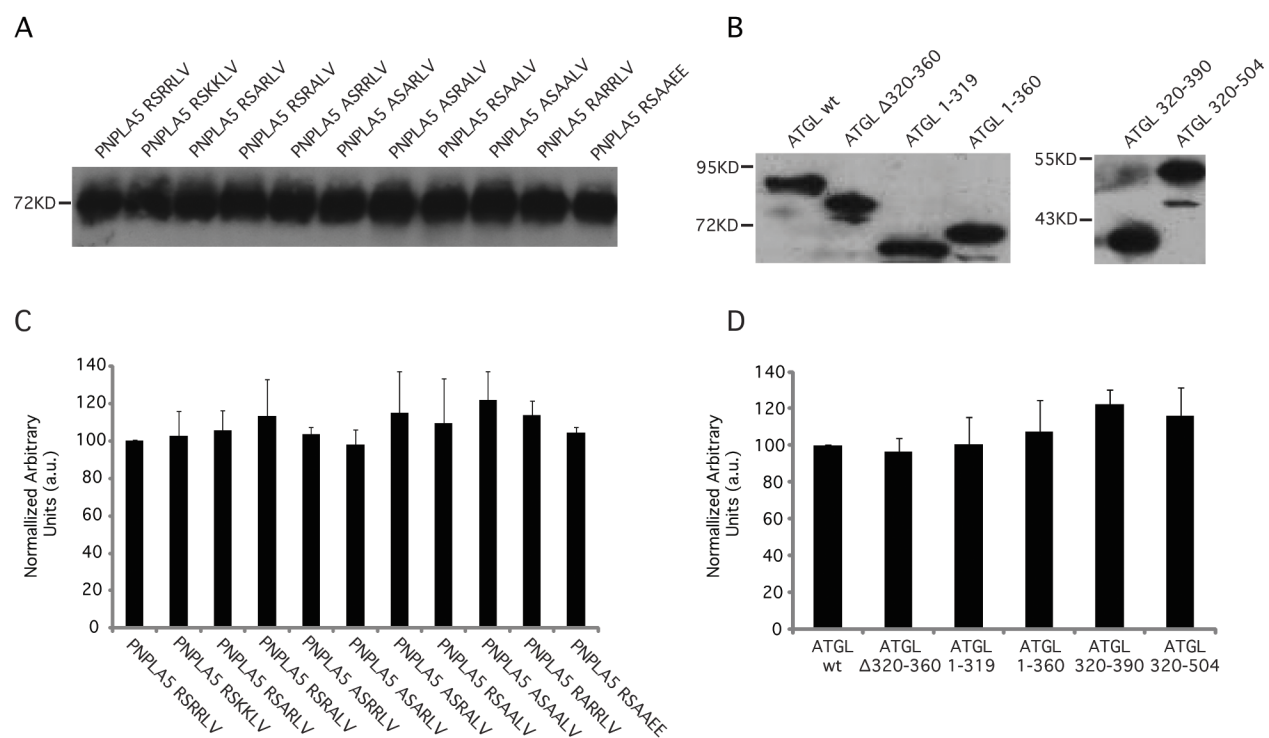

Supplemental Figure S4

Supplement: Figure S4 — Expression levels of PNPLA family LTM constructs. Lysates from cells expressing the indicated GFP-tagged constructs were subjected to western blotting with anti-GFP antibodies. (A) Western blot of GFP-tagged PNPLA5 constructs. (B) Western blot of GFP-tagged ATGL constructs. (C and D) Quantitation of results from A and B, respectively (n = 3) normalized to wildtype expression levels. (PDF) [file pone.0064950.s004.pdf]

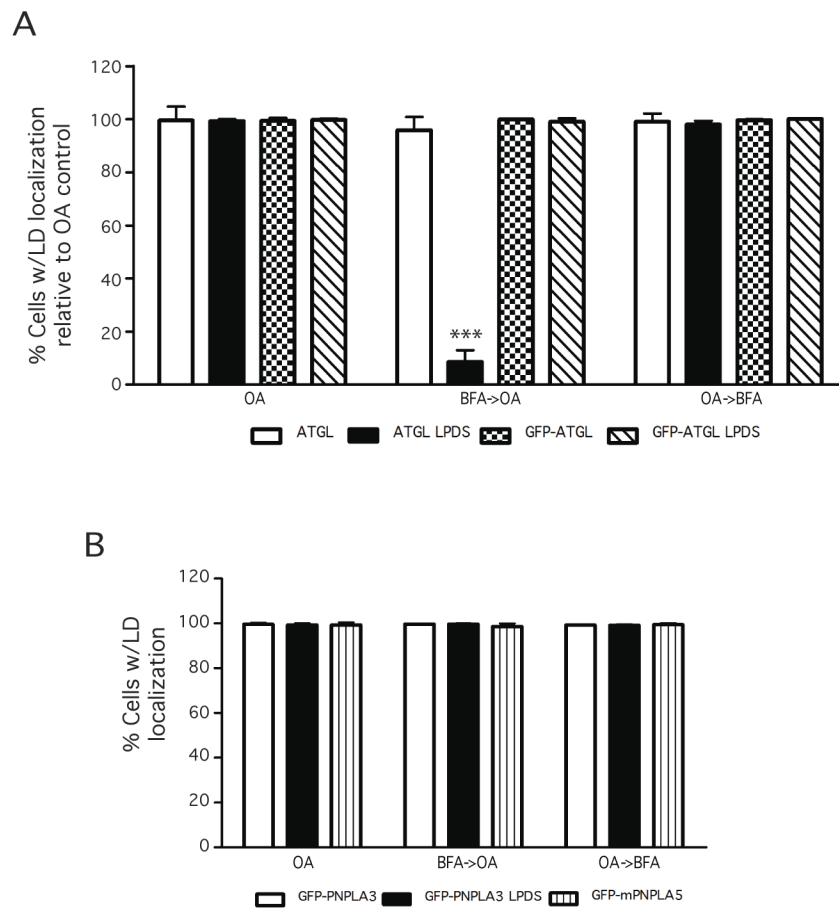

Supplemental Figure S5

Supplement: Figure S5 — Effect of BFA on delivery of PNPLA proteins to LDs. Cells were grown in either 10% FBS or 10% LPDS (lipoprotein deficient serum) for 72 h and then treated with BFA either before or after incubation with OA. To examine delivery to nascent LDs, cells were incubated with 5 μM BFA for 10 min and then fed OA:BSA for 3 h in the continuous presence of BFA. To examine delivery to pre-existing LDs, cells were fed OA:BSA for 3 h to induce LD formation and then incubated with 5 μM BFA for 3 h. Cells were fixed, stained with LipidTox, and examined by fluorescence microscopy. (A) HeLa cells were either transfected to express GFP-ATGL, or stained by immunofluorescence to detect endogenous ATGL. BFA treatment reduced the association of endogenous but not expressed ATGL when cells were grown in LPDS, but it had no effect on expressed or endogenous ATGL when cells were grown in FBS. (B) GFP-tagged PNPLA3 and mPNPLA5 expressed prior to OA incubations were not affected by BFA treatment regardless of serum used. Data are plotted as means ± SE; ≥3 experiments/condition, ≥300 cells counted/experiment. ***indicates p<0.0001 (PDF) [file pone.0064950.s005.pdf]
