# Supplementary material for: Identification of Diverse Lipid Droplet Targeting Motifs in the PNPLA Family of Triglyceride Lipases
Source: PLoS One. 2013 May 31;8(5):e64950. doi: 10.1371/journal.pone.0064950 (PMC3669214; doi:10.1371/journal.pone.0064950)
Supplement: Table SI — Primers used for generating PNPLA truncation constructs. ATGL ATGL 320–504 EcoR1 960 (f): 5′- CCG GAA TTC ATG ACC ACC CTC TCC AAC ATG C-3′ ATGL 320-504 Sal1 1512 (r): 5′- ACG CGT CGA CTC ACA GCC CCA GGG CCC C-3′ ATGL 1-319 EcoR1 1 (f): 5′-CCG GAATTC ATG TAC GAC GCA GAG CG-3′ ATGL 1-319 Sal1 957 (r): 5′-ACGC GTCGAC GGT GTC CAG GAT GCT CTC-3′ ATGL 361-504 EcoR1 1083 (f): 5'-CCG GAA TTC GAG GAC ATC CG-3' ATGL 309-390: ACGC GTCGAC GGG CAG GTG CCT ATGL 1-360 EcoR1 1080 (r): 5'-ACG CGT CGA CGG GAA CGT CGG-3' ATGL Δ320-360 Sal1 957 (r): 5′-CG GAT GTC CTC CAG CAG GTC CGT GGG CTC-3′ ATGL Δ320-360 EcoR1 1080 (f): 5′-CCC ACG GAC CTG CTG GAG GAC ATC CG-3′ PNPLA3 PNPLA3 320-481 EcoR1 960 (f): 5′- CCG GAA TTC CCC AGG CTC GCT ACA GCA CTG -3′ PNPLA3 320-481 Sal1 1443 (r): 5′- TCA CAG ACT CTT CTC TAG TGA AAA ACT GGG -3′ PNPLA5 PNPLA5 1-142 BglII (f): 5′-TAG AGA TCT ATG GGC TTC TTA GAG GAG G-3′ PNPLA5 1-142 KpnI 428 (r): 5′-CAT GGT ACC TTA CTG GAT GAG CTC ATC GCA GGT-3′ PNPLA5 143-285 BglII 429 (f): 5′-TAC AGA TCT GCC TTG GTC TGC ACC TTA TAC-3′ PNPLA5 143-285 KpnI 855 (r): 5′-CAT GGT ACC TTA GCG TTG CTC ACA GGC AGC-3′ PNPLA5 286-429 BglII 858 (f): 5′-TAC AGA TCT TGG AAG GGG GGC CTG TCT C-3″ PNPLA5 286-429 KpnI (r): 5′-CAT GGT ACC TCA GGC CTG GTG GGT GGG-3′ PNPLA5 340-429 BglII 1020 (f): 5′-TAC AGA TCT GTG CTG ACG TAC CTG CTG C-3′ PNPLA5 352-429 BglII 1056 (f): 5′-TAC AGA TCT GAG TAC ATC TAC TTC CGC AGC-3′ PNPLA5 286-352 Kpn1 1056 (r): 5′-CAT GGT ACC TTA GAA GGG CAG TGT GCA GGG-3′ PNPLA5 286-364 Kpn1 1092 (r): 5′-CAT GGT ACC TTA CAC CAC CAA CCT TCT GCT-3′ PNPLA5 286-376 Kpn1 1128 (r): 5′-CAT GGT ACC TTA CAT CCA CCA CAA GTC CGC-3′ mPNPLA5 mPNPLA5 320-432 EcoR1 960 (f): Forward 5′-CCGGAATTCCAGAAGACTGGCCCA-3′ mPNPLA5 320-432 Sal1 1296 (r): 5′-ACGCGTCGACAGTCAGGTACGGT-3′ (DOCX) [file pone.0064950.s006.docx]

**Supplemental Table I**. Primers used for generating PNPLA truncation constructs

ATGL

ATGL 320-504 EcoR1 960 (f): 5’- CCG GAA TTC ATG ACC ACC CTC TCC AAC ATG C-3’

ATGL 320-504 Sal1 1512 (r): 5’- ACG CGT CGA CTC ACA GCC CCA GGG CCC C-3’

ATGL 1-319 EcoR1 1 (f): 5’-CCG GAATTC ATG TAC GAC GCA GAG CG-3’

ATGL 1-319 Sal1 957 (r): 5’-ACGC GTCGAC GGT GTC CAG GAT GCT CTC-3’

ATGL 361-504 EcoR1 1083 (f): 5'-CCG GAA TTC GAG GAC ATC CG-3'

ATGL 309-390: ACGC GTCGAC GGG CAG GTG CCT

ATGL 1-360 EcoR1 1080 (r): 5'-ACG CGT CGA CGG GAA CGT CGG-3'

ATGL ∆320-360 Sal1 957 (r): 5’-CG GAT GTC CTC CAG CAG GTC CGT GGG CTC-3’

ATGL ∆320-360 EcoR1 1080 (f): 5’-CCC ACG GAC CTG CTG GAG GAC ATC CG-3’

PNPLA3

PNPLA3 320-481 EcoR1 960 (f): 5’- CCG GAA TTC CCC AGG CTC GCT ACA GCA CTG -3’

PNPLA3 320-481 Sal1 1443 (r): 5’- TCA CAG ACT CTT CTC TAG TGA AAA ACT GGG -3’

PNPLA5

PNPLA5 1-142 BglII (f): 5’-TAG AGA TCT ATG GGC TTC TTA GAG GAG G-3’

PNPLA5 1-142 KpnI 428 (r): 5’-CAT GGT ACC TTA CTG GAT GAG CTC ATC GCA GGT-3’

PNPLA5 143-285 BglII 429 (f): 5’-TAC AGA TCT GCC TTG GTC TGC ACC TTA TAC-3’

PNPLA5 143-285 KpnI 855 (r): 5’-CAT GGT ACC TTA GCG TTG CTC ACA GGC AGC-3’

PNPLA5 286-429 BglII 858 (f): 5’-TAC AGA TCT TGG AAG GGG GGC CTG TCT C-3”

PNPLA5 286-429 KpnI (r): 5’-CAT GGT ACC TCA GGC CTG GTG GGT GGG-3’

PNPLA5 340-429 BglII 1020 (f): 5’-TAC AGA TCT GTG CTG ACG TAC CTG CTG C-3’

PNPLA5 352-429 BglII 1056 (f): 5’-TAC AGA TCT GAG TAC ATC TAC TTC CGC AGC-3’

PNPLA5 286-352 Kpn1 1056 (r): 5’-CAT GGT ACC TTA GAA GGG CAG TGT GCA GGG-3’

PNPLA5 286-364 Kpn1 1092 (r): 5’-CAT GGT ACC TTA CAC CAC CAA CCT TCT GCT-3’

PNPLA5 286-376 Kpn1 1128 (r): 5’-CAT GGT ACC TTA CAT CCA CCA CAA GTC CGC-3’

mPNPLA5

mPNPLA5 320-432 EcoR1 960 (f): Forward  5’-CCGGAATTCCAGAAGACTGGCCCA-3'

mPNPLA5 320-432 Sal1 1296 (r):   5’-ACGCGTCGACAGTCAGGTACGGT-3’
