# Supplementary material for: Identification of Diverse Lipid Droplet Targeting Motifs in the PNPLA Family of Triglyceride Lipases
Source: PLoS One. 2013 May 31;8(5):e64950. doi: 10.1371/journal.pone.0064950 (PMC3669214; doi:10.1371/journal.pone.0064950)
Supplement: Table S2 — Primers used for generating site-directed mutation of putative LTM. ATGL ATGL AALL (f): 5′-GCT CTG TCC TTC ACC GCC GCC TTG CTG GAG TGG CTG-3′ ATGL AALL (r): 5′-CGG GCA GCC ACA CCA CCA ACG CTG CGC TGC GGA AG-3′ ATGL AAVQ (f): 5′-CAG GTG GAG CTG GCC GCC GTC CAG TCG CTG CC-3′ ATGL AAVQ (r): 5′-GGC AGC GAC TGG ACG GCG GCC AGC TCC ACC TG-3′ ATGL RAAAA (f): 5′-CCT GGT GAT GCG CGC CGC CGC CGC CCT GGG CAGGCA CCT GCC C -3′ ATGL RAAAA (r): 5′-GGG CAG GTG CCT GCC CAG GGC GGC GGC GGC GCG CAT CAC CAG G –3′ ATGL AAAAA (f): 5′-CAG TAC CTG GTG ATG GCC GCC GCC GCC -3′ ATGL AAAAA (r): 5′-GGC GGC GGC GGC CAT CAC CAG GTA CTG-3′ ATGL IAWMA (f): 5′-CCG AGG ACA TCG CT GGA TGG CGG AGC AGA CGG GC-3′ ATGL IAWMA (r): 5′-GCC CGT CTG CTC CGC CAT CCA CGC GAT GTC CTC GG-3′ ATGL AAVQA (f): 5′-GCC GCG TCC AGG CGC TGC CGT CCG T-3′ ATGL AAVQA (r): 5′-ACG GAC GGC AGC ACC TGG ACG CGG C-3′ PNPLA5 AAEE (f): 5′-C TTC CGC AGC GCA GCG GAG GAG GTG TGG CTG CCC GAT GTG CCG-3′ PNPLA5 AAEE (r): 5′-CGG CAC ATC GGG CAG CCA CAC CTC CTC CGG TGC GCT GCG GAA G-3′ PNPLA5 AASAA (f): 5′-CTG ACG TAC CTG CTG CTA GCC GCC TCA GCG GCC TTC GAG TAC ATC-3′ PNPLA5 AASAA (r): 5′-GAT GTA CTC GAA GGC GGC TGA GGC GGC TAG CAG CAG GTA CGT CAG-3′ PNPLA3 PNPLA3 QAAA (f): 5′- CTG CCA TTG CGA TTG TCC AGG CAG CGG CGA CAT GGC TTC CAG -3′ PNPLA3 QAAA (r): 5′-CTG GAA GCC ATG TCG CCG CTG CCT GGA CAA TCG CAA TGG CAG -3′ PNPLA5 PNPLA5 AAEE (f): 5′-C TTC CGC AGC GCA GCG GAG GAG GTG TGG CTG CCC GAT GTG CCG-3′ PNPLA5 AAEE (r): 5′-CGG CAC ATC GGG CAG CCA CAC CTC CTC CGG TGC GCT GCG GAA G-3′ PNPLA5 AASAA (f): 5′-CTG ACG TAC CTG CTG CTA GCC GCC TCA GCG GCC TTC GAG TAC ATC-3′ PNPLA5 AASAA (r): 5′-GAT GTA CTC GAA GGC GGC TGA GGC GGC TAG CAG CAG GTA CGT CAG-3′ PNPLA5 AAAAA (f): 5′-CTG ACG TAC CTG CTG CTA CGG CGG CGA GCG-3′ PNPLA5 AAAAA (r): 5′-GAT GTA CTC GAA GGC CGC TGC GGC GGC TAG-3′ PNPLA5 RSKKLV (f): 5′-CTT CCG CAG CAA AAA GTT GGT GGT GTG GCT GCC-3′ PNPLA5 RSKKLV (r): 5′-GGC AGC CAC ACC ACC AAC TTT TTG CTG CGG AAG-3′ PNPLA5 RSAALV (f): 5′-CTT CCG CAG [file pone.0064950.s007.docx]

**Supplemental Table II.**  Primers used for generating site-directed mutation of putative LTM

ATGL

ATGL AALL (f): 5’-GCT CTG TCC TTC ACC GCC GCC TTG CTG GAG TGG CTG-3’

ATGL AALL (r): 5’-CGG GCA GCC ACA CCA CCA ACG CTG CGC TGC GGA AG-3’

ATGL AAVQ (f): 5’-CAG GTG GAG CTG GCC GCC GTC CAG TCG CTG CC-3’

ATGL AAVQ (r): 5’-GGC AGC GAC TGG ACG GCG GCC AGC TCC ACC TG-3’

ATGL RAAAA (f): 5’-CCT GGT GAT GCG CGC CGC CGC CGC CCT GGG CAGGCA CCT GCC C -3’

ATGL RAAAA (r): 5’-GGG CAG GTG CCT GCC CAG GGC GGC GGC GGC GCG CAT CAC CAG G –3’

ATGL AAAAA (f): 5’-CAG TAC CTG GTG ATG GCC GCC GCC GCC -3’

ATGL AAAAA (r): 5’-GGC GGC GGC GGC CAT CAC CAG GTA CTG-3’

ATGL IAWMA (f): 5’-CCG AGG ACA TCG CT GGA TGG CGG AGC AGA CGG GC-3’

ATGL IAWMA (r): 5’-GCC CGT CTG CTC CGC CAT CCA CGC GAT GTC CTC GG-3’

ATGL AAVQA (f): 5’-GCC GCG TCC AGG CGC TGC CGT CCG T-3’

ATGL AAVQA (r): 5’-ACG GAC GGC AGC ACC TGG ACG CGG C-3’

PNPLA5 AAEE (f): 5’-C TTC CGC AGC GCA GCG GAG GAG GTG TGG CTG CCC GAT GTG CCG-3’

PNPLA5 AAEE (r): 5’-CGG CAC ATC GGG CAG CCA CAC CTC CTC CGG TGC GCT GCG GAA G-3’

PNPLA5 AASAA (f): 5’-CTG ACG TAC CTG CTG CTA GCC GCC TCA GCG GCC TTC GAG TAC ATC-3’

PNPLA5 AASAA (r): 5’-GAT GTA CTC GAA GGC GGC TGA GGC GGC TAG CAG CAG GTA CGT CAG-3’

PNPLA3

PNPLA3 QAAA (f): 5’- CTG CCA TTG CGA TTG TCC AGG CAG CGG CGA CAT GGC TTC CAG -3’

PNPLA3 QAAA (r): 5’-CTG GAA GCC ATG TCG CCG CTG CCT GGA CAA TCG CAA TGG CAG -3’

PNPLA5

PNPLA5 AAEE (f): 5’-C TTC CGC AGC GCA GCG GAG GAG GTG TGG CTG CCC GAT GTG CCG-3’

PNPLA5 AAEE (r): 5’-CGG CAC ATC GGG CAG CCA CAC CTC CTC CGG TGC GCT GCG GAA G-3’

PNPLA5 AASAA (f): 5’-CTG ACG TAC CTG CTG CTA GCC GCC TCA GCG GCC TTC GAG TAC ATC-3’

PNPLA5 AASAA (r): 5’-GAT GTA CTC GAA GGC GGC TGA GGC GGC TAG CAG CAG GTA CGT CAG-3’

PNPLA5 AAAAA (f): 5’-CTG ACG TAC CTG CTG CTA CGG CGG CGA GCG-3’

PNPLA5 AAAAA (r): 5’-GAT GTA CTC GAA GGC CGC TGC GGC GGC TAG-3’

PNPLA5 RSKKLV (f): 5’-CTT CCG CAG CAA AAA GTT GGT GGT GTG GCT GCC-3’

PNPLA5 RSKKLV (r): 5’-GGC AGC CAC ACC ACC AAC TTT TTG CTG CGG AAG-3’

PNPLA5 RSAALV (f): 5’-CTT CCG CAG CGC AGC GTT GGT GGT GTG GCT GCC CG-3’

PNPLA5 RSAALV (r): 5’-CGG GCA GCC ACA CCA CCA ACG CTG CGC TGC GGA AG-3’

PNPLA5 RSARLV (f): 5’-CTT CCG CAG CGC AAG GTT GGT GGT GTG GC-3’

PNPLA5 RSARLV (r): 5’-GCC ACA CCA CCA ACC TTG CGC TGC GGA AG-3’

PNPLA5 RSRALV (f): 5’-CTT CCG CAG CAG AGC GTT GGT GGT GTG GC-3’

PNPLA5 RSRALV (r): 5’-GCC ACA CCA CCA ACG CTC TGC TGC GGA AG-3’

PNPLA5 RARRLV (f): 5’-CAT CTA CTT CCG CGC CAG AAG GTT GGT GGT G-3’

PNPLA5 RARRLV (r): 5’-CAC CAC CAA CCT TCT GGC GCG GAA GTC GAT G-3’

PNPLA5 ASARLV (f): 5’-CAT CTA CTT CGC CAG CGC AAG GTT GGT GGT GTG G-3’

PNPLA5 ASARLV (r): 5’-CCA CAC CAC CAA CCT TGC GCT GGC GAA GTA GAT G-3’

PNPLA5 ASRALV (f): 5’- CAT CTA CTT CGC CAG CAG AGC GTT GGT GGT GTG G-3’

PNPLA5 ASRALV (r): 5’-CCA CAC CAC CAA CGC TCT GCT GGC GAA GTA GAT G-3’

PNPLA5 ASRRLV (f): 5’-GAG TAC ATC TAC TTC GCC AGC AGA AGG T-3’

PNPLA5 ASRRLV (r): 3’-ACC TTC TGC TGG CGA AGT AGA TGT ACT C-3’

PNPLA5 ASAALV (f): 5’-GTA CAT CTA CTT CGC CAG CGC AGC GTT GGT GG-3’

PNPLA5 ASAALV (r): 5’-CCA CCA ACG CTG CGC TGG CGA AGT AGA TGT AC-3’

Brummer Lipase

BL AAAAA (f): 5’-CCA CGC CAT GGC GGC AGC AGC GGC-3’

BL AAAAA (r): 5’-GGC GCT GCT GCC GCC ATG GCG TGG-3’

BL AAAAANA (f): 5’-GGC AGC AGC GGC AAA TGC ATT CAC GCT CTA TGA C-3’

BL AAAAANA (r): 5’-GTC ATA GAG CGT GAA TGC ATT TGC CGC TGC TGC C-3'
