# Supplementary material for: Identification of Diverse Lipid Droplet Targeting Motifs in the PNPLA Family of Triglyceride Lipases
Source: PLoS One. 2013 May 31;8(5):e64950. doi: 10.1371/journal.pone.0064950 (PMC3669214; doi:10.1371/journal.pone.0064950)
Supplement: Table S3 — Primers used to generate PNPLA4 fusion constructs. PNPLA4-cterm ATGL (f): 5′-ATG AAG CAC ATC AAC CTA TCA TTT GCA GCG-3′ (r): 5′-TCC CGG CAG CTG CGA GTA ATG TTC AAA CCA ATT TTC TTT AAG-3′ (f): 5′-CTT AAA GAA AAT TGG TTT GAA GAT TAC TCG CAG CTG CCG GGA-3′ (r): 5′-TCA CAG CCC CAG GGC CCC GAT-3′ EcoR1 (f): 5′-CCG GAA TTC ATG AAG CAC ATC AAC CTA TCAT-3′ Sal1 (r): 5′- ACG CGT CGA CTC ACA GCC CCA GGG CCC C-3′ PNPLA4-cterm PNPLA3 (f): 5′-ATG AAG CAC ATC AAC CTA TCA TTT GCA GCG-3′ (r): 5′-CTT AAA GAA AAT TGG TTT GAA CCC AGG CTC GCT ACA GCA CTG-3′ (f): 5′-CTT AAA GAA AAT TGG TTT GAA CCC AGG CTC GCT ACA GCA CTG-3′ (r): 5′-TCA CAG ACT CTT CTC TAG TGA AAA ACT GGG-3′ EcoR1 (f): 5′-CCG GAA TTC ATG AAG CAC ATC AAC CTA TCAT-3′ Sal1 (r): 5′-ACG CGT CGA CTC ACA GAC TCT TCT CTA GTG A-3′ PNPLA4-cterm PNPLA5 (f): 5′-ATG AAG CAC ATC AAC CTA TCA TTT GCA GCG-3′ (r): 5′-GTT GAG AGA CAG GCC CCC CTT TTC AAA CCA ATT TTC TTT AAG-3′ (f): 5′-CTT AAA GAA AAT TGG TTT GAA AAG GGG GGC CTG TCT CTC AAC-3′ (r): 5′-TCA GGC CTG GTG GGT GGG CCC-3′ EcoR1 (f): 5′-CCG GAA TTC ATG AAG CAC ATC AAC CTA TCAT-3′ Sal1 (r): 5′-ACG CGT CGA CTC AGG CCT GGT-3′ (DOCX) [file pone.0064950.s008.docx]

**Supplemental Table III**. Primers used to generate PNPLA4 fusion constructs

PNPLA4-cterm ATGL

(f): 5’-ATG AAG CAC ATC AAC CTA TCA TTT GCA GCG-3’

(r): 5’-TCC CGG CAG CTG CGA GTA ATG TTC AAA CCA ATT TTC TTT AAG-3’

(f): 5’-CTT AAA GAA AAT TGG TTT GAA GAT TAC TCG CAG CTG CCG GGA-3’

(r): 5’-TCA CAG CCC CAG GGC CCC GAT-3’

EcoR1 (f): 5’-CCG GAA TTC ATG AAG CAC ATC AAC CTA TCAT-3’

Sal1 (r): 5’- ACG CGT CGA CTC ACA GCC CCA GGG CCC C-3’

PNPLA4-cterm PNPLA3

(f): 5’-ATG AAG CAC ATC AAC CTA TCA TTT GCA GCG-3’

(r): 5’-CTT AAA GAA AAT TGG TTT GAA CCC AGG CTC GCT ACA GCA CTG-3’

(f): 5’-CTT AAA GAA AAT TGG TTT GAA CCC AGG CTC GCT ACA GCA CTG-3’

(r): 5’-TCA CAG ACT CTT CTC TAG TGA AAA ACT GGG-3’

EcoR1 (f): 5’-CCG GAA TTC ATG AAG CAC ATC AAC CTA TCAT-3’

Sal1 (r): 5’-ACG CGT CGA CTC ACA GAC TCT TCT CTA GTG A-3’

PNPLA4-cterm PNPLA5

(f): 5’-ATG AAG CAC ATC AAC CTA TCA TTT GCA GCG-3’

(r): 5’-GTT GAG AGA CAG GCC CCC CTT TTC AAA CCA ATT TTC TTT AAG-3’

(f): 5’-CTT AAA GAA AAT TGG TTT GAA AAG GGG GGC CTG TCT CTC AAC-3’

(r): 5’-TCA GGC CTG GTG GGT GGG CCC-3’

EcoR1 (f): 5’-CCG GAA TTC ATG AAG CAC ATC AAC CTA TCAT-3’

Sal1 (r): 5’-ACG CGT CGA CTC AGG CCT GGT-3’
